# Reversible Photocontrol of Dopaminergic Transmission in Wild-Type Animals

**DOI:** 10.3390/ijms231710114

**Published:** 2022-09-04

**Authors:** Carlo Matera, Pablo Calvé, Verònica Casadó-Anguera, Rosalba Sortino, Alexandre M. J. Gomila, Estefanía Moreno, Thomas Gener, Cristina Delgado-Sallent, Pau Nebot, Davide Costazza, Sara Conde-Berriozabal, Mercè Masana, Jordi Hernando, Vicent Casadó, M. Victoria Puig, Pau Gorostiza

**Affiliations:** 1 Institute for Bioengineering of Catalonia (IBEC), the Barcelona Institute for Science and Technology, 08028 Barcelona, Spain; 2 Biomedical Research Networking Center in Bioengineering, Biomaterials and Nanomedicine (CIBER-BBN), 28029 Madrid, Spain; 3 Department of Pharmaceutical Sciences, University of Milan, 20133 Milan, Italy; 4 Hospital del Mar Medical Research Institute (IMIM), Barcelona Biomedical Research Park, 08003 Barcelona, Spain; 5 Department of Biochemistry and Molecular Biomedicine, Faculty of Biology, Institute of Biomedicine, University of Barcelona, 08028 Barcelona, Spain; 6 Department of Biomedical Sciences, Faculty of Medicine and Health Sciences, Institute of Neuroscience, University of Barcelona, IDIBAPS, CIBERNED, 08036 Barcelona, Spain; 7 Department of Chemistry, Autonomous University of Barcelona (UAB), 08193 Cerdanyola del Vallès, Spain; 8 Catalan Institution for Research and Advanced Studies (ICREA), 08010 Barcelona, Spain

**Keywords:** azobenzene, behavior, brainwave, dopamine, GPCR, in vivo electrophysiology, optogenetics, optopharmacology, photochromism, photopharmacology, photoswitch, zebrafish

## Abstract

Understanding the dopaminergic system is a priority in neurobiology and neuropharmacology. Dopamine receptors are involved in the modulation of fundamental physiological functions, and dysregulation of dopaminergic transmission is associated with major neurological disorders. However, the available tools to dissect the endogenous dopaminergic circuits have limited specificity, reversibility, resolution, or require genetic manipulation. Here, we introduce azodopa, a novel photoswitchable ligand that enables reversible spatiotemporal control of dopaminergic transmission. We demonstrate that azodopa activates D_1_-like receptors in vitro in a light-dependent manner. Moreover, it enables reversibly photocontrolling zebrafish motility on a timescale of seconds and allows separating the retinal component of dopaminergic neurotransmission. Azodopa increases the overall neural activity in the cortex of anesthetized mice and displays illumination-dependent activity in individual cells. Azodopa is the first photoswitchable dopamine agonist with demonstrated efficacy in wild-type animals and opens the way to remotely controlling dopaminergic neurotransmission for fundamental and therapeutic purposes.

## 1. Introduction

**Figure 1:**
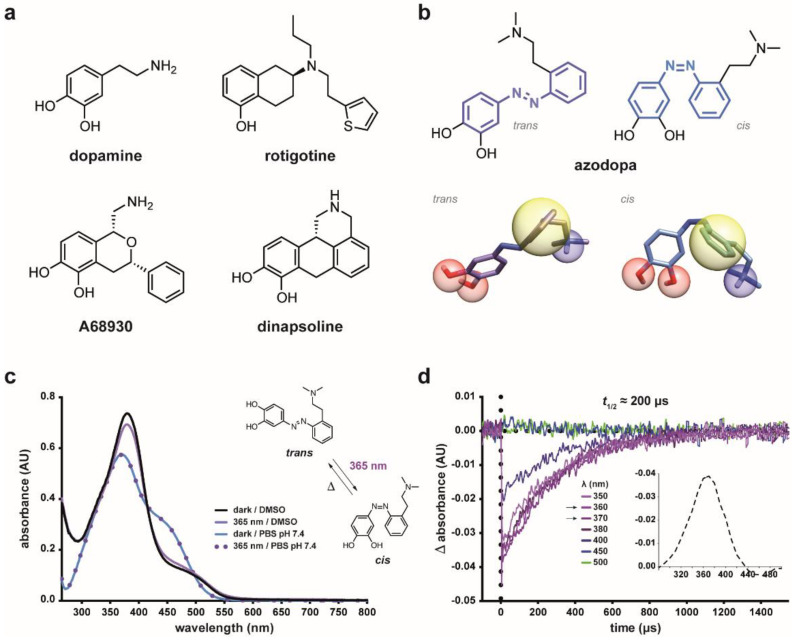
**Design, structure, and photochromism of azodopa.** (**a**) Chemical structure of dopamine and representative (semi)rigidified derivatives: rotigotine (nonselective agonist), A68930 (D_1_ agonist), and dinapsoline (D_1_ agonist). (**b**) 2D and 3D chemical structure of the photochromic dopamine ligand azodopa (*trans* and *cis* isomers). Essential pharmacophoric features for D_1_ receptor binding are highlighted: blue spheres represent cationic site points and *H*-bond donors, i.e., the protonated amino function that can form a salt bridge and a hydrogen bond with Asp103 and Ser107, respectively; red spheres represent *H*-bond acceptors, i.e., the hydroxyl groups of the catechol ring that can interact with Ser198, Ser199, and Ser202; yellow spheres represent hydrophobic elements, i.e., the aromatic ring that can form π–π interactions with Phe288 and Phe289. The mutual position and orientation of such pharmacophoric features in the receptor-bound conformation should affect binding affinity and efficacy of the ligands. (**c**) Photochromic behavior of azodopa (50 µM) studied with steady-state spectroscopy in aqueous (PBS, pH 7.4) and organic (DMSO) solutions. As lifetimes of *cis*-hydroxyazobenzenes are very short in polar protic solvents, no changes in the absorption spectrum of aqueous solutions of azodopa could be observed after illumination with 365 nm light (3 min). (**d**) Photochromic behavior of azodopa (30 µM) investigated by transient absorption spectroscopy in water (only representative traces are shown for the sake of clarity; see Figure S7 for the full experiment). Transient absorption time traces were measured at different wavelengths upon excitation of *trans*-azodopa with a 5 ns pulsed laser at λ = 355 nm (3 mJ/pulse energy) and 25 °C. Thermal relaxation half-life of the *cis* isomer (200 μs) was estimated by applying an exponential one-phase decay model (GraphPad Prism 6). Inset: Transient absorption spectrum of *trans*-azodopa upon pulsed irradiation at λ = 355 nm recorded at t = 0 μs. X-values represent wavelength (nm), Y-values represent ∆A (arbitrary units, AU).

Dopamine receptors (DARs) are members of the class A G protein-coupled receptor (GPCR) family and are prominent in the vertebrate central nervous system (CNS). Their primary endogenous ligand is the catecholaminergic neurotransmitter dopamine, a metabolite of the amino acid tyrosine. Among the many neuromodulators used by the mammalian brain to regulate circuit function and plasticity, dopamine (Figure 1a) stands out as one of the most behaviorally powerful [1,2]. Dopaminergic neurons are critically involved in diverse vital CNS functions, including voluntary movement, feeding, reward, motivation, sleep, attention, memory, and cognition. The extracellular concentration of dopamine oscillates following day/night cycles and plays important physiological roles in the regulation of olfaction, retinal function [3], and circadian rhythms [4]. Disentangling these diverse components of DAR signaling and dopaminergic transmission is an unmet need both of basic research and medicine, because their abnormal function leads to complex medical conditions such as Parkinson’s and Huntington’s diseases, schizophrenia, attention deficit hyperactivity disorder, Tourette’s syndrome, drug abuse, and addiction [1]. To date, five subtypes of DARs have been cloned: D_1_, D_2_, D_3_, D_4_, and D_5_. Based on their coupling to either G_αs,olf_ proteins or G_αi/o_ proteins, which, respectively, stimulate or inhibit the production of the second messenger cAMP, DARs are classified as D_1_-like receptors (D_1_, D_5_) or D_2_-like receptors (D_2_, D_3_, D_4_). However, both classes are known to signal through multiple pathways. Targeting these receptors using specific agonists and antagonists allows to modulate dopaminergic transmission and dopamine-dependent functions. Indeed, hundreds of compounds interfering with the dopaminergic system have been developed, and many of them are clinically used to treat various disorders. They also constitute pharmacological tools to study the role of dopamine in synaptic and neural circuits [5,6] as well as the mechanisms underlying dopamine-related debilitating conditions [7]. However, conventional ligands cannot differentiate among specific neuronal sub-populations in heterogeneous brain regions where multiple neuronal subtypes exist, thus potentially activating DARs that mediate distinct or even opposing physiological functions [2]. For this reason, the lack of circuit selectivity is a confounding element in basic research and likely cause of the poor safety as well as efficacy of many dopaminergic drugs. Hence, methods to activate DARs noninvasively with high spatiotemporal resolution are required both for research and therapeutic purposes.

Pursuing a traditional pharmacological approach would hardly pay off in such a physiological scenario, because a drug generally affects its target in multiple CNS regions at once and its effect is slowly reversible. Light is an unparalleled input signal to noninvasively manipulate biological systems in precisely designated patterns, and photopharmacology [8,9,10], which relies on molecular photoswitches to regulate bioactive compounds, has already been successful in GPCRs [11,12,13,14,15,16], ion channels [17,18], and enzymes [19,20]. The possibility of using light as an external stimulus to manipulate specific populations of dopaminergic neurons has generated enormous interest in neurobiology since the invention of optogenetics [21,22], in particular to elucidate the basis of complex behavioral and cognitive processes [23,24]. However, the achievements of optogenetics rely on the overexpression of exogenous proteins that lack critical aspects of endogenous GPCR signaling, including their native ligand binding sites, downstream molecular interactions, and other elements that can affect receptor dynamics. Thus, current optogenetic tools may provide partial, or sometimes inaccurate, insights into biological processes. In addition, the application of genetic manipulation techniques to human subjects is still importantly hampered by safety (possible immune responses against the gene transfer), regulatory, and economic issues. Complementary approaches to address some of these limitations have been proposed. Trauner, Isacoff, and collaborators developed light-gated DARs through a combined chemical–genetic method in which a weakly photoswitchable ligand was tethered via a maleimide–thiol conjugation to a genetically engineered cysteine residue at the target receptor in order to improve photocontrol [25]. They also used a genetically targeted membrane anchor to tether a dopaminergic ligand via SNAP-tag labeling [15]. Unlike the exogenous optogenetics tools described above, light-gated receptors bear a single-point mutation and provide a nearly physiological study system, but they still require gene delivery and overexpression. Genetics-free methods have also been described. For instance, Etchenique, Yuste, and collaborators developed a caged dopamine compound based on ruthenium–bipyridine chemistry and used it to activate dendritic spines with two-photon excitation [26]. More recently, Gmeiner et al. described two caged DAR antagonists that can serve as valuable tools for light-controlled blocking of D_2_/D_3_ receptors [27]. However, notwithstanding its virtues, uncaging is an irreversible chemical process, while a photopharmacological modulation based on reversible, byproduct-free molecular photoswitches has important advantages in vivo. Noteworthy in this regard is the work published by König and collaborators, who developed a set of photochromic small molecules by incorporation of dithienylethenes and fulgides into known dopamine receptor ligands [28]. Two of those compounds, named **29** and **52**, showed an interesting isomer-dependent (open vs. closed form) efficacy at activating D_2_ receptors, although neither in situ photoswitching nor in vivo validation of their effects were reported. We present here the design, synthesis, and photopharmacological characterization of the first chemical tool that enables the reversible photocontrol of native dopamine receptors in wild-type animals. Notably, it can be used to separate the retinal component of dopaminergic neurotransmission in zebrafish and manipulate brain waves in mice.

## 2. Results

### 2.1. Design Strategy and Synthesis

Aryl azo compounds, especially azobenzenes, have emerged as the photoswitches of choice in photopharmacology because of their physical and chemical properties, which make them especially suitable for biological applications [29,30]. An elegant strategy for the incorporation of an azobenzene into a bioactive ligand [11] relies on the isosteric replacement of the two-atom linker between the two aromatic rings with a diazene unit (–N=N–) [11,20,31,32,33], which entails minimal perturbation of pharmacophore and drug-like properties, thus accounting for the success of this so-called azologization approach [31]. However, to the best of our knowledge, this strategy is not applicable to any of the known dopamine agonists, since the scaffold of an isomerizable aryl azo compound is not directly conceivable in their structure.

In the quest for a freely diffusible drug-like dopaminergic photoswitch, we noticed that most agonists (especially to D_1_ receptors, see Figure 1a) are rigid or semi-rigid, conformationally restrained structures in which essential pharmacophoric features are held in their mutual position [34]. Indeed, two main routes can be identified in the early development of dopamine agonists, namely, the rigidification of the dopamine molecule and the dissection of apomorphine, one of the first potent dopamine agonists to be found [35]. Dopamine rigidification led to the discovery of potent agonists, whereas the apomorphine de-rigidification generally reduced its efficacy [36,37]. It stands then to reason that governing the geometry of such structures would enable the control of their biological effects. This could be achieved by “building” upon a semi-rigid and photoisomerizable molecular frame the structural elements required for DAR activation and using light as external control signal. This approach can be likened to a “photoswitch decoration”, in which pharmacophore groups are introduced into the structure of a molecular photoswitch to design a light-controlled bioactive compound. Common features in the dopamine agonist pharmacophore model are the following: (I) a cationic site point (an amino group) that forms a salt bridge with an aspartic acid residue in the receptor’s third transmembrane helix (TM3); (II) one or two hydrogen bond acceptor/donor sites (e.g., hydroxy groups), that interact with serine residues in TM5; and (III) an aromatic ring system that takes part in π–π interactions with hydrophobic residues in TM6 [38,39,40]. On these grounds, we devised a photoswitchable dopamine agonist by decorating an azobenzene molecule with two hydroxyl substituents (II) on one phenyl ring, and an amino group (I) connected through a short linker to the other phenyl ring (III) (Figure 1b). This molecule, that we named azodopa, carries the main DAR binding determinants and enables to change their relative position upon photoisomerization (Figure 1b). Azodopa was synthesized in two steps via an azo-coupling reaction between commercially available 2-(2-(dimethylamino)ethyl)aniline and 1,2-dihydroxybenzene (see supplementary Scheme S1 and Supplementary Materials, SM, for detailed synthetic procedures and physicochemical characterization).

### 2.2. Photochemical Characterization

An essential condition for azodopa to function as a photoswitchable dopamine ligand is that it photoisomerizes between two different configurations (*trans* and *cis*). We first characterized it by steady-state UV–Vis absorption spectroscopy (Figure 1c), which revealed an absorption maximum at 370 nm in aqueous solution (PBS, pH 7.4) and at 380 nm in DMSO. It is known that the thermal *cis*-to-*trans* isomerization of 4-hydroxyazobenzenes follows a very fast kinetics in polar protic solvents via solvent-assisted proton transfer tautomerization, whereas it proceeds more slowly in aprotic and nonpolar solvents [41]. In agreement, no changes in the absorption spectrum of azodopa could be detected with a conventional UV–Vis spectrophotometer upon illumination at 365 nm in aqueous buffer because of the short lifetime of its *cis* isomer in this medium, likely because of the presence of a non-negligible concentration of azonium cation at physiological pH and/or the formation of hydrogen-bonded complexes with the solvents that accelerate the thermal *cis*-to-*trans* isomerization via tautomerization, and only a small change could be recorded in a polar but aprotic solvent such as DMSO, where the tautomerization is still possible, although less pronounced (Figure 1c). No measurements were performed in nonpolar solvents (e.g., toluene) because of the very poor solubility of our compound in this kind of media. We next determined the optimal isomerization wavelength and lifetime of *cis*-azodopa by means of transient-absorption spectroscopy with ns-time resolution. Upon pulsed excitation of azodopa in aqueous media with UV and violet light, an instantaneous and remarkable decrease of the absorption signal was detected, which can be ascribed to the depletion of the *trans* ground electronic state due to the photoinduced formation of the corresponding *cis* isomer (Figure 1d) [41,42]. The initial absorption value was quickly recovered because of the fast thermal *cis*-to-*trans* back-isomerization which restores the initial concentration of the *trans* isomer with an estimated half-life of about 200 μs (Figure 1d). Thus, since conversion to the *trans* form occurs almost immediately after turning the light off, we performed all biological experiments under continuous illumination. Overall, our absorption spectroscopy studies showed that azodopa undergoes *trans*-to-*cis* photoisomerization upon illumination with UV and violet light and it spontaneously reverts to its full *trans* isomer in a fraction of a millisecond once the light is switched off. Other mechanisms, such as excited-state intramolecular proton transfer, could also play a role. In any case, azodopa should allow fast regulation of DAR activity using a single illumination source to induce *trans*-to-*cis* isomerization.

### 2.3. In Vitro Pharmacology

**Figure 2:**
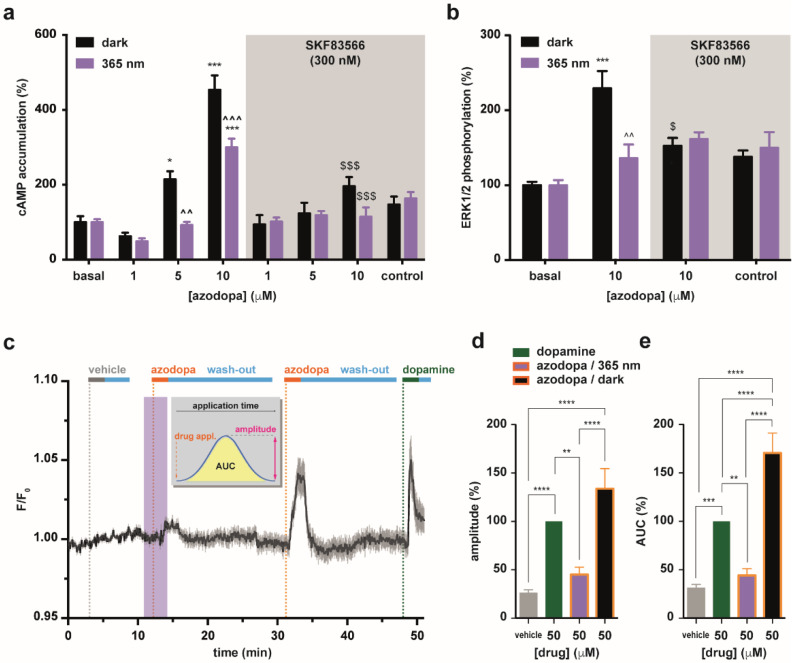
**In vitro pharmacological characterization of azodopa.** (**a**) Effect on D_1_-mediated adenylyl cyclase activation. cAMP accumulation experiments in HEK-293T cells transiently transfected with D_1_ and treated with different concentrations of azodopa, in the dark (black bars) or under illumination (purple bars), in the presence (gray area) or not (white area) of a D_1_-like receptor antagonist (SKF83566). Values are represented in percentage vs. basal levels of cAMP. Data are mean ± S.E.M. (6 experiments performed in quadruplicate). Statistical differences were analyzed by two-way ANOVA followed by Tukey’s post hoc test (*** *p* < 0.001 vs. basal; * *p* < 0.05 vs. basal; ^^^ *p* < 0.001 vs. dark; ^^ *p* < 0.01 vs. dark; $$$ *p* < 0.001 vs. controls non-pretreated with the antagonist). (**b**) Effect on D_1_-mediated ERK1/2 activation. ERK1/2 phosphorylation was determined in HEK-293T cells transiently transfected with D_1_ and treated with different concentrations of azodopa, in the dark (black bars) or under illumination (purple bars), in the presence (gray area) or not (white area) of a D_1_-like antagonist (SKF83566). Values are represented in percentage vs. basal levels of ERK1/2 phosphorylation. Data are mean ± S.E.M. (3 or 4 experiments performed in triplicate or quadruplicate). Statistical differences were analyzed by two-way ANOVA followed by Tukey’s post hoc test (*** *p* < 0.001 vs. basal; ^^ *p* < 0.01 vs. dark; $ *p* < 0.05 vs. controls non-pretreated with the antagonist). (**c**–**e**) Effect on D_1_-mediated intracellular calcium release compared to dopamine. (**c**) Real-time calcium imaging response (averaged traces, black line, n = 24 cells) in HEK-293T cells co-expressing D_1_ receptors and R-GECO1 as calcium indicator. Traces were recorded upon direct application of azodopa (50 µM, orange bars) in the dark (white area) and under illumination (purple area). Shadow represents “± S.E.M.”. Gray and green bars indicate the application of vehicle (control) and dopamine (reference agonist), respectively. Light blue bars indicate wash-out periods. See example frames and raw data traces of individual cells in supplementary Figure S10, and supplementary Video S1 for the entire movie. Two values of the calcium responses generated by azodopa were calculated (Origin 8 software) and compared: the peak amplitude ΔF/F_0_ (**d**), calculated as the difference between the maximal and the minimal intensity of each response (**** *p* < 0.0001 for vehicle vs. dopamine; **** *p* < 0.0001 for vehicle vs. azodopa/dark; **** *p* < 0.0001 for azodopa/365 nm vs. azodopa/dark; ** *p* = 0.035 for dopamine vs. azodopa/365 nm), and the area under the curve (*AUC*) (**e**), calculated as the integral over the entire application time of vehicle or drugs (**** *p* < 0.0001 vehicle vs. dopamine; **** *p* < 0.0001 for vehicle vs. azodopa/dark; **** *p* < 0.0001 for dopamine vs. azodopa dark; **** *p* < 0.0001 for azodopa/365 nm vs. azodopa/dark; *** *p* = 0.001 for vehicle vs. dopamine; ** *p* = 0.0025 for dopamine vs. azodopa/365 nm). Data are mean ± S.E.M. (n = 40 cells from 3 independent experiments). Data were normalized over the maximum response obtained with the saturating concentration of dopamine (50 μM) and were analyzed by one-way ANOVA followed by Tukey’s post hoc test for statistical significance. All statistical analyses (panels (**a**,**b**,**d**,**e**)) were performed with GraphPad Prism 6.

We next tested the effects of azodopa on D_1_-like receptors, for which freely diffusible photoswitchable agonists have not been reported. First, we evaluated the binding affinity of azodopa in mammalian D_1_-like and D_2_-like receptors using competitive radioligand binding experiments performed either in the dark or under illumination at 365 nm (see SM for details) [43,44]. Azodopa displayed a good capacity to bind to D_1_-like and D_2_-like receptors, with higher affinity in the *trans* configuration in both cases. In particular, for D_1_-like receptors, we calculated an almost fourfold decrease in affinity at 365 nm (*K*_d_^dark^ = 600 nM, *K*_d_^365 nm^ = 2200 nM) (supplementary Figure S8 and Table S1). Since the activation of D_1_-like receptors promotes cyclic adenosine monophosphate (cAMP) formation and phosphorylation by ERK1/2, we also investigated the ability of azodopa to behave as a D_1_-like agonist by studying cAMP accumulation (Figure 2a) and ERK1/2 phosphorylation (Figure 2b) in cells overexpressing human D_1_ receptors, both in the dark and under continuous illumination at 365 nm (to compare the “full *trans*” vs. the “*cis*-enriched” form, respectively). Intracellular cAMP accumulation was measured in HEK-293T cells transiently transfected with D_1_ receptors in a time-resolved fluorescence resonance energy transfer (TR-FRET) assay. We found that azodopa induced cAMP accumulation in a dose- and light-dependent manner (Figure 2a). In particular, we observed substantial differences between the two forms at 5 μM and 10 μM, with the *trans* form being significantly more effective at inducing cAMP production than the *cis*-enriched form at the same concentrations. When cells were co-treated with the D_1_-like antagonist SKF83566 (300 nM), the effect of azodopa was largely reduced or even disappeared, indicating that azodopa activates D_1_ receptors. In cAMP assays performed in non-transfected HEK-293T cells used as negative control, azodopa (10 μM), dopamine (1 μM), and SKF38393 (300 nM) did not produce any effect (Figure S9). ERK1/2 phosphorylation was measured in HEK-293T cells transiently transfected with D_1_ by Western blot analysis using phospho-ERK1/2 antibody. The application of azodopa (10 μM) promoted ERK1/2 phosphorylation to an extent that was significantly different between the two conditions (dark vs. light). The full *trans* form displayed an efficacy 3.6-fold greater than the *cis*-enriched form (Figure 2b), in agreement with the observations in cAMP accumulation assays. Again, co-treatment of the cells with SKF83566 abolished azodopa responses, showing that the effects observed were mediated by D_1_. Although we did not thoroughly characterize the pharmacological activity profile, azodopa displayed D_1_-mediated photoactivity (blocked by SKF83566) also in zebrafish (see later).

It is known that D_1_ receptors are also linked to other second messenger systems. These include receptor-mediated activation of phospholipase C (G_q_ coupling) to generate inositol 1,4,5-trisphosphate (IP_3_) which participates in phosphoinositide turnover and calcium-regulated signaling pathways in the brain [45]. IP_3_ receptors are mainly located in the endoplasmic reticular membrane where IP_3_ can mobilize Ca^2+^ from intracellular stores [45]. Thus, as a complementary method to characterize azodopa in vitro, we performed Ca^2+^-imaging experiments in HEK-293T cells co-expressing D_1_ receptors and R-GECO1 as calcium indicator, and used dopamine as a control (Figure 2c–e, supplementary Figures S10 and S11, supplementary Video S1). Azodopa (50 μM) was applied to the cells both in the dark and under illumination with UV light. Dopamine (50 μM) was tested as reference agonist. Robust increases of intracellular calcium were observed by the application of azodopa in the dark (pure *trans* isomer), whereas only weak increases were recorded when azodopa was applied under illumination (Figure 2c and Figure S10). Calcium responses were quantified and compared by peak amplitude and area under the curve (AUC). The analysis of these parameters showed that *trans*-azodopa stimulates the release of intracellular calcium (similar to dopamine) and this effect is abolished under illumination. In this experiment, *trans*-azodopa displayed significantly higher efficacy than dopamine (Figure 2d,e). No responses were observed in control experiments in HEK cells (n = 25) not expressing D_1_, thus confirming that the calcium oscillations were elicited by a specific interaction at this receptor (Figure S11). Moreover, we verified that *trans*-azodopa fully recovers its efficacy after long (60 min) exposure to 365 nm light, demonstrating that its effects are reversibly photodependent and are not due to an irreversible photodegradation of the molecule (Figures S20 and S21). Our results in calcium imaging experiments suggest that *trans*-azodopa activates the G_q_/phospholipase C pathway, in addition to the canonical G_s_/adenylyl cyclase pathway already investigated. Such intriguing behavior has been described also for other dopamine agonists [45].

Taken as a whole, the results from our in vitro experiments illustrated in Figure 2 show that *trans*-azodopa is a full agonist at D_1_-like receptors and that its effects can be partially or completely switched off with light. The reduction of azodopa efficacy under illumination can be attributed to the photoisomerization process which decreases the partial concentration of the *trans* isomer at the target receptor and/or disrupts the ligand interaction(s) at the binding pocket.

### 2.4. Behavioral Effects in Zebrafish Larvae

The promising photopharmacological profile of azodopa prompted to use it in vivo to modulate dopaminergic neurotransmission. For that purpose, we designed a behavioral assay to record and quantify the locomotor activity of living animals as a function of drug concentration and illumination. It is known that dopamine plays a pivotal role in motor control in humans, as it activates striatal direct pathway neurons that directly project to the output nuclei of the basal ganglia through D_1_ receptors, whereas it inhibits striatal indirect pathway neurons that project to the external pallidum through D_2_ receptors [46]. We chose zebrafish larvae (*Danio rerio*) as animal model for our experiments because of their transparency, which facilitates the delivery of light, and their morphological, genetic, and behavioral similarity to higher vertebrates [10,47,48,49,50,51,52]. Indeed, a functional nervous system is established after only 4–5 days of embryonic development in zebrafish, enabling them to perform complex behaviors such as swimming and exploratory activity. In particular, the major dopaminergic pathways in mammals are also represented in the zebrafish brain, and homologous receptors for most of the mammalian subtypes have been identified in these animals. Humans and zebrafish share 100% of the amino acids in the binding site for D_1_ and D_3_ receptors, and 85–95% for D_2_ and D_4_ receptors, and generally similar effects are observed for dopaminergic ligands in zebrafish and in mammals [51]. As a rule of thumb, dopamine receptor agonists increase the locomotor activity, whereas antagonists decrease it [53]. However, disentangling the action of drugs on the multiple components of dopaminergic transmission (including different brain regions [2], the regulation of retinal function [3], and of circadian rhythms [4]) constitutes an unmet need of pharmacology and medicine. Therefore, we set to test azodopa in vivo and to take advantage of photocontrolling dopaminergic responses.

Zebrafish larval movements were tracked using a ZebraBox device for automated behavioral recording. Zebrafish larvae at 6 days post-fertilization (6 dpf) were randomly divided into control (vehicle) and treatment groups (with azodopa added to water). Each individual was placed in a separate well of a 96-well plate. Our setup allowed exposing the animal to controlled cycles of dark and 365 nm UV illumination, using the following protocol: dark (20 min, for adaptation), UV light (30 s), dark (20 min), and then four cycles of UV light (30 s) and dark (5 min) (see SM for details and supplementary Video S2). In order to identify alterations in behavior, we measured multiple properties of locomotor activity to determine the activity level [54]. In particular, we focused on fast movements and measured swimming distances and duration of fast swimming, defined as speed ≥6 mm·s^−1^.

**Figure 3:**
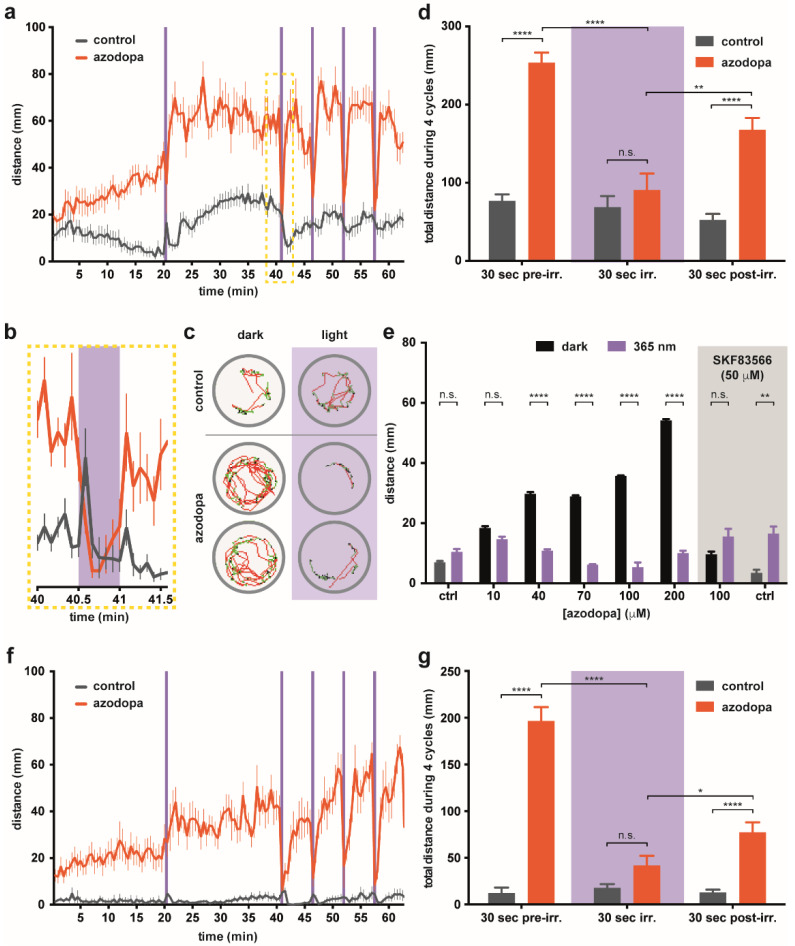
**Behavioral effects of azodopa in zebrafish.** (**a**–**e**) Experiments with normal zebrafish. (**a**) Swimming activity (distance/time) in larvae exposed to vehicle (control, gray line) or 100 μM azodopa (treatment, orange line) in the dark (white areas) or under illumination with 365 nm light (purple bars). Data are mean ± S.E.M. (n = 11–12 individuals/group). (**b**) Representative time frame (40–41.5 min) of the swimming activity integrated every 5 s, showing how the effect of azodopa can be completely shut down upon illumination. The spike of activity observed for the control group upon illumination represents the startle response to the light stimulus. (**c**) Exemplary trajectories of individual larvae in one well containing the vehicle and two wells containing 100 μM azodopa in the dark (40–40.5 min) and under illumination (40.5–41 min). Green lines and red lines indicate slow and fast swimming periods, respectively. The remarkable and light-dependent difference in behavior between untreated and azodopa-treated fish can be appreciated by observing these trajectories and the supplementary Video S2. (**d**) Quantification of the total distances swum by the control group (vehicle) and the treatment group (100 μM azodopa) during 4 consecutive cycles of illumination (30 s) and dark (30 s before and after illumination). Data are mean ± S.E.M. (n = 11–12 individuals/group) and were analyzed by two-way ANOVA followed by Tukey’s post hoc test (**** *p* < 0.0001; ** *p* = 0.0037). (**e**) Dose–response study of azodopa (white area) and effect of a co-administered D_1_-like antagonist (gray box). Different groups of larvae were exposed to increasing concentrations of azodopa. For quantification, the average distance swum by each group during the last 4 consecutive dark–light cycles (30 s integration) was considered. The graph shows that *trans*-azodopa (black bars) increases the fish locomotor activity in a dose-dependent manner, but its effects are abolished by the co-administration of a potent and selective D_1_-like antagonist (SKF83566, 50 μM). Data are mean ± S.E.M. (n = 12 individuals/group) and were analyzed by two-way ANOVA followed by Sidak’s post hoc test (**** *p* < 0.0001; ** *p* = 0.0063). (**f**,**g**) Experiments with blinded zebrafish. (**f**) Swimming activity (distance/time) in larvae exposed to vehicle (control, gray line) or 100 μM azodopa (treatment, orange line) in the dark (white areas) or under illumination with 365 nm light (purple bars). Data are mean ± S.E.M. (n = 12 individuals/group). (**g**) Quantification of the total distances swum by the control group (vehicle) and the treatment group (100 μM azodopa) during 4 consecutive cycles of illumination (30 s) and dark (30 s before and after illumination). Data are mean ± S.E.M. (n = 12 individuals/group) and were analyzed by two-way ANOVA followed by Tukey’s post hoc test (**** *p* < 0.0001; * *p* = 0.0232). All statistical analyses (panels (**d**,**e**,**g**)) were performed with GraphPad Prism 6.

We first studied a wide concentration range (spanning from 1 nM to 1 mM azodopa) to determine if behavioral effects could be observed without signs of acute toxicity. No significant differences in locomotor activity were observed between the control group and the treatment groups up to a concentration of 10 μM, neither in the dark nor upon illumination (Figure S12). A strong increase in swimming activity was recorded at 1 mM, but the effect ceased after 30 min, possibly because the fish were exhausted. We observed the most interesting alterations of the behavioral profile at 100 μM (Figure 3a–d and Figure S12). The changes in the swimming activity over time for this group and the control group are plotted in Figure 3a (full experiment represented in Figure S12; see also Video S2). We detected a progressive increase in activity for 30–40 min and relatively small photoresponses (e.g., at 20 min in Figure 3a) which is often due to the slow uptake of the drug in the fish [14]. After this period, animals treated with 100 μM azodopa displayed a high swimming activity in the dark that was sustained for the whole duration of the experiment, and that was abolished during each period of UV illumination (30 s bouts between minute 40 and 60 in Figure 3a). These results agree with the intracellular signaling photoresponses observed in vitro and confirm a *trans*-on/*cis*-off dopamine agonist profile. An averaged time course (between 40–41.5 min, integrated every 5 s) of the swimming activity is magnified in Figure 3b. Representative trajectories of individual fish in wells containing the vehicle or 100 μM azodopa in the dark (40–40.5 min) and under illumination (40.5–41 min) are shown in Figure 3c, where green and red lines indicate slow (<6 mm·s^−1^) and fast (≥6 mm·s^−1^) swimming periods, respectively (see Figure S13 for the entire plate and SM for experimental details and analysis). Figure 3d shows the quantification and statistical analysis of the total distances swum by the control group and the treatment group (100 μM azodopa) during the last four cycles, namely, once the maximum effect of the drug was reached and maintained (30 s pre-illumination, 30 s illumination, and 30 s post-illumination periods). Fish treated with azodopa covered a distance three times longer than the control group during the same dark periods, and this effect was blocked under illumination. Interestingly, we also noticed a reduction in swimming activity during the first 30 s of dark after each illumination pulse in the treatment group, likely because the fish needed some time to recover the maximum level of activity. The differences between the total distances swum by the treatment group during the 30 s periods of dark (pre- or post-illumination) and the 30 s periods of illumination were also statistically significant (Figure 3d).

Next, we sought to verify whether the effect of azodopa on zebrafish locomotor activity is dose-dependent by testing a range of concentrations around 100 μM. For our analysis, we averaged the distance swum by each group during the last four consecutive dark–light cycles (30 s integration). We observed that azodopa increased the swimming activity in the dark in a dose-dependent manner, while the effect under 365 nm illumination was smaller at all concentrations and yielded a weak dose dependence (Figure 3e; see also Figure S14 for a representation of the activity profile at all concentrations). Moreover, we observed that co-application of the D_1_-like antagonist SKF83566 (50 μM) abolished the behavioral effects produced by azodopa at 100 μM in the dark, and restored zebrafish activity to control levels (Figure 3e and Figure S15). These experiments allow to exclude the participation of adrenergic receptors in the photoresponses in vivo [14], and support the hypothesis that they are mediated by D_1_-like rather than D_2_-like receptors (SKF83566 is thousand-fold more potent in the formers). However, they cannot rule out the involvement of 5-HT_2_ receptors, as the antagonist binds them with only 20-fold weaker potency.

To distinguish between the contribution of visual responses to (1) the changes in fish locomotion and (2) the dopaminergic modulation with azodopa, we repeated the experiments with blinded zebrafish larvae. The zebrafish retina contains four different cone photoreceptor subtypes (UV, S, M, L), each one defined by the expression of specific opsins that confer a particular wavelength-sensitivity. UV cones express SWS1, an opsin with peak sensitivity in the UV range (λ_max_ = 354 nm) [55]; therefore, the removal of functional UV cones can be used to suppress UV-dependent behaviors [56]. Blinded zebrafish larvae were obtained via a noninvasive blinding technique that induces photoreceptor apoptosis while preserving the rest of the retina (see SM for details) [57]. They were remarkably inactive and unresponsive to illumination (Figure 3f,g). The activity profile of azodopa-treated blinded zebrafish was qualitatively similar to the one observed with azodopa-treated normal zebrafish (Figure 3a,f respectively). Azodopa (100 μM) produced a remarkable increase of the swimming activity of blind larvae for the entire experiment during the dark periods, and this effect was abolished upon illumination. Quantification of the total distances swum by the control group and the treatment group (100 μM azodopa) during the last four dark/light cycles confirmed our observations: the locomotion of blinded animals could be significantly photoswitched with azodopa. The overall decrease in swimming activity of blinded zebrafish (Figure 3f,g) compared to normal zebrafish (Figure 3a–e) is due to the induced blindness, which reduces their exploratory tendencies. This phenomenon is more pronounced in the control-blinded group, which is almost immobile, and makes the effect of azodopa on locomotion appear even stronger: azodopa-treated blinded larvae covered a total distance 16 times longer (during pre-illumination periods) and 6 times longer (during post-illumination periods) than the one swum by control animals (Figure 3g).

The fact that azodopa can elicit behavioral photoresponses in blinded zebrafish and that they have similar magnitude to those in normal fish show that retinal photoreceptors are not directly involved in the observed change in locomotion upon illumination. Instead, photoresponses must be attributed to other dopaminergic circuits in the CNS (present both in blinded and normal larvae) that are effectively placed under the control of light with azodopa. Interestingly, the time course of photoresponses does display differences between blinded and normal larvae, and these must be ascribed to the presence of visual inputs in normal animals. The most outstanding one is the recovery of locomotion after turning off UV light in the presence of azodopa, which is significantly faster in normal zebrafish larvae than in blinded ones (see four cycles in Figure 3a,f, and quantification in supplementary Figure S16). Thus, azodopa enables time-resolved behavioral experiments that contain unique information about the dopaminergic modulation of retinal function [3], and that will be further investigated with spatially-resolved neuronal activity maps.

Overall, behavioral responses in zebrafish larvae agree with our previous findings in vitro and confirm that azodopa is a reversible photoswitchable dopaminergic agonist displaying dose-dependent locomotor effects. In addition, we demonstrated that zebrafish larvae, previously exposed to azodopa and dark–light cycles, recovered normal swimming behavior after washout (see supplementary Figure S22 and SM), and were still alive about 48 h after the experiment. The robust photocontrol of behavior and the apparent absence of acute toxicity encouraged us to test the potential of azodopa in a mammalian model.

### 2.5. Electrophysiological Recordings in Anesthetized Mice

**Figure 4:**
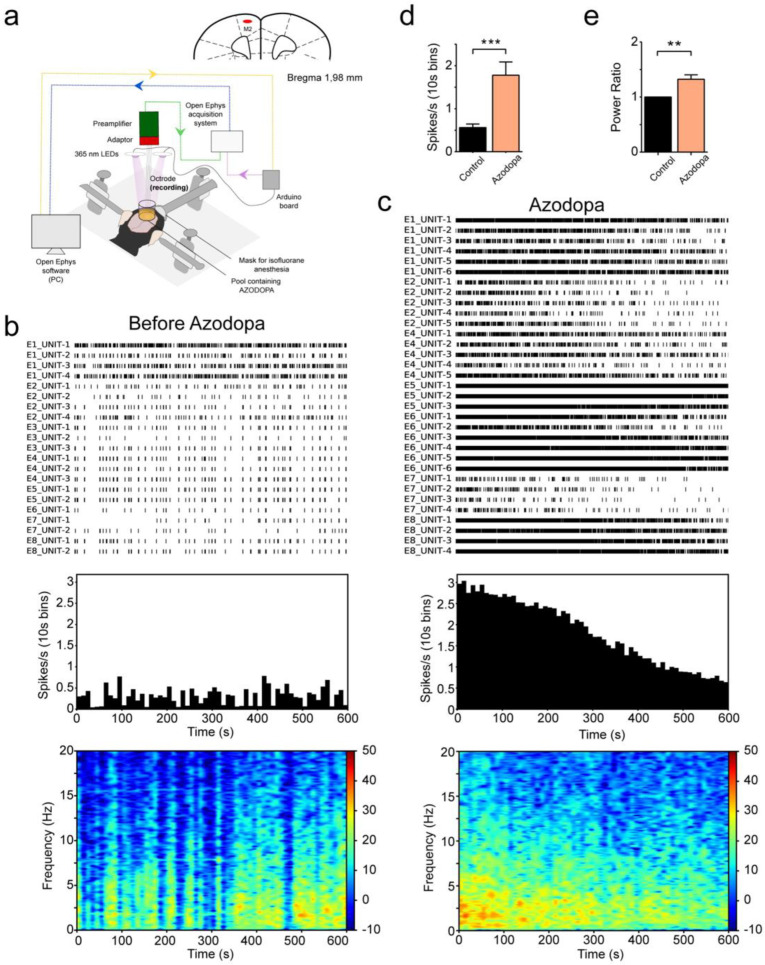
**Effect of cortical administration of *trans*-azodopa on electrophysiological recordings in anesthetized mice.** (**a**) Animals were anesthetized with isoflurane and placed in a stereotaxic apparatus. A craniotomy was drilled above the secondary motor cortex (M2) and an octrode was inserted in the superficial layers. Analogic signals were bandpass filtered and digitized by a preamplifier, amplified by an Open Ephys data acquisition system (green arrow), and finally visualized and recorded in a PC (blue arrow). Neural activity was recorded during baseline conditions and after administration of *trans*-azodopa on the cortical surface (3 μM concentration in 10 μL volume). See the Supplementary Materials for further details of the setup and supplementary Figures S17–S19 for the effect of illumination on azodopa. (**b**) Neural activity during baseline conditions in Mouse 1. Raster plot of spiking activity in the cortex of one mouse for 10 min. Each row depicts the spiking activity of a single neuron (unit), each tick representing an action potential. We used arrays of 8 electrodes (octrodes) in each animal, from which several units could be recorded. Neurons are labeled by their electrode number (E1 to E8). Firing rates were stable and followed the UP and DOWN slow fluctuations typical of anesthesia. The quantification of firing rates and average time–frequency spectrogram of the power of neural oscillations (n = 8 electrodes) are shown below. (**c**) Neural activity after the administration of 3 μM *trans*-azodopa in Mouse 1 (zero indicates the time of administration). Azodopa boosted the firing rate of neurons and increased the power of neural oscillations. (**d**) Azodopa increased spiking activity of cortical neurons. Mean firing rate of neurons before and after the administration of *trans*-azodopa. Data are mean ± S.E.M. (n = 44 neurons during baseline vs. 43 neurons after azodopa in two mice) and were analyzed with an unpaired *T*-test (*** *p* = 0.0002). (**e**) Azodopa increased the power (1–10 Hz) of neural oscillations in the two animals. Due to the large differences in the baseline power of the two mice, we normalized the power to its baseline for visualization purposes only. Data are mean ± S.E.M. (n = 16 channels per condition from two mice) and were analyzed with a paired *T*-test (** *p* = 0.0011).

We studied whether azodopa modulates neural activity in the cortex of mice. For such experiments, we developed a custom setup that combined in vivo electrophysiological recordings and the possibility to illuminate with 365 nm LEDs. The Open Ephys data acquisition system was used to record neural activity via an octrode (four two-wire stereotrode array) inserted in the superficial layers of the secondary motor cortex (M2) with a large craniotomy that allowed exposure of cortical tissue (Figure 4a).

We first investigated the effects of azodopa in the absence of light in two mice anesthetized with isoflurane (Mouse 1 and Mouse 2, Figure 4b–e). In each animal, we recorded spiking activity of individual neurons (single unit activity, SUA) and local field potentials (LFPs) in M2 before and after the administration of *trans*-azodopa. We quantified mean firing rates (spikes per second) and LFP power from 1 to 10 Hz. Transient oscillatory signals observed in LFPs reflect the summed synchronized activity of neural networks and are also called neural oscillations. Neural oscillations between 1 and 10 Hz have been associated with cognitive processing during alertness and slow waves during slow wave sleep and anesthesia (i.e., UP and DOWN states). The experiments started after cortical activity was stable for at least 10 min. Then, we recorded baseline neural activity for 10 more minutes. During this period, slow fluctuations of neural activity were observed both at the single-neuron and LFP levels that were associated with UP and DOWN states typically observed during anesthesia (Figure 4b). Later, 10 μl of *trans*-azodopa at 3 µM concentration were administered manually on the surface of the cortex with a standard pipette. Azodopa increased neural activity few seconds after its administration. The effects were transient in many neurons and the general activity remained elevated for at least 5 min (Figure 4c). There was a boost in the firing rate of individual neurons that no longer followed the UP and DOWN cycles. In addition, more neurons could be identified after *trans*-azodopa administration (21 neurons during baseline and 33 neurons after azodopa in mouse 1; see also supplementary Figure S17). In fact, in mouse 2, we isolated 23 neurons during baseline and only 10 neurons after *trans*-azodopa because spiking activity was so elevated that spikes from different neurons co-occurred altering the shape of the waveforms and thus prevented their classification. Corresponding with the increased spiking activity of neurons, the power of oscillatory activities augmented, although moderately compared to changes in firing rate (Figure 4c, bottom panel). Statistical analyses confirmed significant increases of firing rate and 1–10 Hz power when combining the two animals (Figure 4d,e).

We next investigated the effects of azodopa under 365 nm illumination in two more mice anesthetized with isoflurane (Mouse 3 and Mouse 4, supplementary Figures S17–S19). In each mouse, we first injected 0.5 mL of saline and later 0.5 mL 3 µM azodopa in consecutive experiments. Solutions were administered with an infusion pump over the course of one minute to avoid environmental noise and to allow electrophysiological recordings during the administration. Moreover, drug administration was conducted under illumination so that azodopa penetrated cortical tissue in an inactive form. Then, we illuminated the motor cortex with five consecutive cycles of one minute of darkness (OFF) and one minute of light (ON) (supplementary Figure S17). Here, we focused on differential spiking activity during light and dark cycles because azodopa displayed a stronger effect than on the power of neural oscillations in the previous experiments (Figure 4).

After the injection of saline, the mean firing rate of neurons remained similar to baseline levels, and neural activity slightly increased during the light cycles compared to the dark cycles (supplementary Figure S17). In the presence of azodopa, neural activity increased as in direct application experiments (Figure 4) and some neurons showed rapid and reversible changes in their firing patterns during the ON and OFF light cycles. Interestingly, some of these neurons decreased their spiking activity during the light periods (DARK-ON) and others increased it (LIGHT-ON; supplementary Figures S18 and S19). Although these heterogeneous effects of light cancelled each other out in the global average (supplementary Figure S17c), manual pooling of single unit recordings yielded significant differences in neural activity between dark and light conditions (supplementary Figure S19). Since dopamine receptors are expressed both in excitatory and inhibitory neurons of the frontal cortex, we hypothesize that DARK-ON neurons may correspond to those expressing D_1_-like receptors, which decrease their firing rates when azodopa is deactivated by light, and LIGHT-ON neurons may be cells affected indirectly by network effects driven by DARK-ON neurons.

Overall, our first in vivo studies in anesthetized mice indicate that *trans*-azodopa exerts excitatory actions on brain cortical microcircuits, as reflected by the increased spiking and oscillatory activities. The effects of azodopa were transient; the firing rate of many neurons was increased for few minutes, while in others the elevated firing lasted more than 10 min. This could be due to the diffusion (and therefore dilution) of azodopa within cortical tissue over time, and possibly to its metabolic washout and reuptake by synaptic terminals [58]. The increase of cortical activity by *trans*-azodopa is consistent with our results from functional studies in cell cultures and suggests that *trans*-azodopa may also act as a dopamine agonist in the mouse brain [59]. The general excitatory action of azodopa was modulated by UV light in individual neurons, some of which revealed a decrease in spiking activity while others increased it. These heterogeneous responses to D_1_-like receptor activation in wild-type animals bear physiological relevance and are currently being studied.

## 3. Discussion

Understanding the dopaminergic system dynamics is a central question in neurobiology and neuropharmacology. In fact, DARs are involved in the modulation of fundamental physiological functions such as voluntary movement, motivation, cognition, emotion, reward, and neuroendocrine secretion, among others, and a dysregulation of the dopaminergic transmission is unavoidably associated with major psychiatric and neurological disorders. However, the available techniques to dissect neuronal circuits and their role in pathological conditions have several shortcomings. Electrical stimulation lacks cellular specificity and conventional pharmacological manipulation lacks temporal and spatial resolution [60]. Optogenetic tools allow the modulation of specific neural circuit elements with millisecond precision, but are limited by non-uniform expression of the optogenetic actuators and generation of non-physiological patterns of activity throughout the targeted population of neurons. Here, we introduce a novel photopharmacological agonist that enables reversible spatiotemporal control of intact dopaminergic pathways in vivo. We show that azodopa triggers DAR-mediated cAMP accumulation and ERK1/2 phosphorylation as well as phospholipase C activation in its *trans* configuration, and its efficacy can be reduced or switched off under illumination. Accordingly, azodopa allows the reversible photocontrol of zebrafish motility on a timescale of seconds, increasing the swimming activity exclusively in the *trans* active state. Azodopa can be bath-applied, does not require microinjection or genetic manipulation, and is compatible with high-throughput behavioral screening in wild-type or transgenic fish, and with other treatments including pharmacological ones. For example, the inactivity of blinded larvae under control conditions can be risen to levels comparable to normal (untreated) animals by adding *trans*-azodopa in the water without illumination. In addition, locomotion is reduced to control levels upon photoisomerization to the *cis* form, which suggests that azodopa might be used to interfere with extracellular dopamine/melatonin cycles in the retina involved in circadian rhythms [3]. Furthermore, the intriguing observation that DAR activation by azodopa *cis-trans* isomerization (dark relaxation) produces faster behavioral responses in normal fish than in blinded ones offers new opportunities to interrogate the dopaminergic modulation of retinal circuits with spatiotemporal, pharmacological, and cell-type specificity [61]. Our results thus complement recently reported genetic–photopharmacological agonists [15] and photopharmacological antagonists [25], and open the way to dissect dopaminergic neurotransmission in intact animals. Characterizing in detail the pharmacological profile and safety of azodopa was not the aim of this work, but experiments with zebrafish in the presence of a selective antagonist suggested that D_1_-like receptors are the main mediators of the photocontrolled behavior.

*trans*-Azodopa also exerts excitatory actions on brain cortical microcircuits at firing rates and frequency bands relevant for behavior in mice [62]. In agreement with our in vitro studies, azodopa induced a general increase in neural excitability, although the time course and light dependence of the responses was heterogeneous in individual neurons. We identified cortical neurons with light-ON and dark-ON patterns, which is expected when administering in the intact brain an agonist of dopamine receptors that are expressed both in excitatory and inhibitory neurons. These responses must be characterized further, but it must be noted that isoflurane anesthesia produces profound inhibitory effects on brain activity. The activity of neural networks may be more difficult to modulate under anesthesia than during alertness, which prompts to evaluate the effects of azodopa under light/dark regimes in awake mice.

From a photochromic point of view, azodopa displays a short half-life of thermal relaxation, which is useful in neurobiology since a single wavelength of light allows to rapidly toggle the photoswitch between its two isomers. However, azodopa requires UV light for deactivation, which is normally undesired in photopharmacology because of poor tissue penetration and potential phototoxicity. Moreover, azodopa is active in the most thermodynamically stable configuration (*trans*). Although there are clinical conditions that might take advantage of a dark-active drug that can be deactivated on demand (e.g., to reduce levodopa-induced dyskinesia in Parkinson’s disease) [63], light-activatable compounds are generally preferred for research and therapeutic purposes. Hence, new dopaminergic photoswitches must be developed that are active in the less thermodynamically stable configuration [64] and photoisomerize with visible [65] or infrared light [66,67] in order to unleash their full potential as photopharmacological tools.

In summary, azodopa is a photochromic activator of endogenous dopamine receptors that does not require genetic manipulation, and is the first photoswitchable dopaminergic agonist with demonstrated efficacy in vivo in intact wild-type animals, including mammals. This ligand allows analyzing the different components of the dopaminergic circuitry and is a breakthrough in developing new photoswitchable drugs potentially useful to manage neurological conditions, including movement disorders and addiction.

## Data Availability

The datasets generated during and/or analyzed during the current study are available from the corresponding author on reasonable request.

## Supplementary Materials

The following supporting information can be downloaded at: https://www.mdpi.com/article/10.3390/ijms231710114/s1, References [68,69] are cited in the supplementary materials.
