# Supplementary material for: Reversible Photocontrol of Dopaminergic Transmission in Wild-Type Animals
Source: Int J Mol Sci. 2022 Sep 4;23(17):10114. doi: 10.3390/ijms231710114 (PMC9456102; doi:10.3390/ijms231710114)
Supplement: Supplementary file 1 [file ijms-23-10114-s001.zip › captions to Video S1 and Video S2.pdf]

**Video S1. Calcium imaging time lapse sequence of HEK293 cells co-transfected with dopamine $D_1$ receptors (unlabeled) and GECO1 calcium fluorescent reporter (red).** Cells were subsequently challenged with vehicle, *cis*-azodopa, *trans*-azodopa, and dopamine (as indicated in the label) with perfusion of washout solution in between. Several cells (indicated with arrows) respond to dopamine and *trans*-azodopa (which act as non-conventional $D_1$ receptor agonists triggering intracellular calcium release) but not to *cis*-azodopa or vehicle (demonstrating that azodopa activity is reversibly photoswitchable in wildtype receptors). See fluorescence response traces of individual cells, quantification, statistical analysis, and selected frames of this video in Figures 2CDE, Suppl. Figures S10 and S11.

**Video S2. Effects of azodopa on larval zebrafish locomotion in different light conditions.** A video clip of the experiment represented in Figure 3 (panels a–d) and Figures S12 and S13, corresponding to the last four dark-light cycles (from minute 37 to minute 60). To help the viewer perceive the photoswitchable effect of azodopa, two representative wells from the 'ctrl' (vehicle) group and the '100 μM' (azodopa) group are circled in yellow. All fish at 100 μM show a remarkable increase of the locomotor activity in the dark, while they almost immediately stop swimming upon illumination (365 nm light, overlay text in the video) and for the whole duration of the illumination period. After an initial period of hyperactivity (about 25 min, not shown in this clip), larvae exposed to 1 mM azodopa become physically exhausted and thus appear to be hypoactive in this second part of the experiment.
