# Supplementary material for: Reversible Photocontrol of Dopaminergic Transmission in Wild-Type Animals
Source: Int J Mol Sci. 2022 Sep 4;23(17):10114. doi: 10.3390/ijms231710114 (PMC9456102; doi:10.3390/ijms231710114)
Supplement: Supplementary file 1 [file ijms-23-10114-s001.zip › supplementary materials.pdf]

SUPPLEMENTARY MATERIALS

TABLE OF CONTENTS

1. Chemical Synthesis & Characterization
2. Photochemical Characterization
3. Radioligand Binding Assays
4. cAMP Accumulation Assays
5. ERK Phosphorylation Assays
6. Ca^{2+} Imaging Assays
7. Behavioral Assays in Zebrafish
8. Electrophysiological Recordings in Mice
9. Azodopa Resistance to Photodegradation
10. Recovery of Normal Swimming Behavior of Zebrafish Larvae after Washout
11. Additional References

1. Chemical Synthesis & Characterization

All chemicals and solvents are from commercial suppliers and used without purification. Reactions were monitored by thin layer chromatography (TLC: EMD/Millipore, silica gel 60 on aluminium support, layer thickness: 200 µm, particle size: 10-12 µm) by visualisation under 254 and/or 365 nm lamp. Nuclear magnetic resonance spectrometry (NMR): Varian-Mercury 400 MHz. Chemical shifts (δ) are reported in parts per million (ppm) against the reference compound tetramethylsilane using the signal of the residual non-deuterated solvent [Methanol-d_4 δ = 3.31 ppm (^{1}H), δ = 49.00 ppm (^{13}C)]. High-performance Liquid Chromatography (HPLC) apparatus: Waters Alliance 2695 separation module coupled to Waters 2996 photodiode detector (PDA) with MassLynx 4.1 software for data acquisition; XSelect CSH C18 OBD Preparative Column (130 Å, 5 µm, 10 mm X 150 mm); mobile phase: water w/0.1% formic acid (solvent A) and acetonitrile w/0.1% formic acid (solvent B); elution method: flow 3 mL/min, gradient 0.0-1.0 min, 0% B; 1.0−7.0 min, 0−100% B; 7.0−8.0min, 100% B; 8.0−9.0 min, 100−0% B; 9.0−10.0 min, 0% B; runtime 10 min. Mass spectroscopy (MS) apparatus: Waters ACQUITY QDa detector (single quad mass detector) equipped with an electrospray ionization (ESI) interface. Spectra have been scanned between 100 and 1000 Da with values every 0.1 seconds and peaks are given as mass/charge (*m*/*z*) ratio. High resolution mass spectrometry analyses were performed with a LTQ-FT Ultra Mass Spectrometer (Thermo Scientific) with NanoESI positive ionization. A sample of the final compound was reconstituted in 100 µL of MeOH and diluted 1/100 with CH_3CN/H_2O/formic acid (50:50:1) for MS analysis. The sample was introduced by direct infusion (Automated Nanoelectrospray). The NanoMate (Advion BioSciences, Ithaca, NY, USA) aspirated the samples from a 384-well plate (protein Lobind) with disposable, conductive pipette tips, and infused the samples through the nanoESI Chip (which consists of 400 nozzles in a 20x20 array) towards the mass spectrometer. Spray voltage was 1.70 kV, delivery pressure 0.50 psi and *m*/*z* range 50-2000 Da. Data were acquired with Xcalibur software, vs.2.0SR2 (ThermoScientific). Elemental composition from experimental exact mass monoisotopic value was obtained with a dedicated algorithm integrated in Xcalibur software. Data are reported as mass-to-charge ratio (*m*/*z*) of the corresponding positively charged molecular ion.

Synthetic Procedure. Azodopa was prepared in fair yield (about 63%) via an azo coupling reaction between freshly diazotized 2-(2-(dimethylamino)ethyl)aniline and 1,2-dihydroxybenzene (**Scheme S1**).

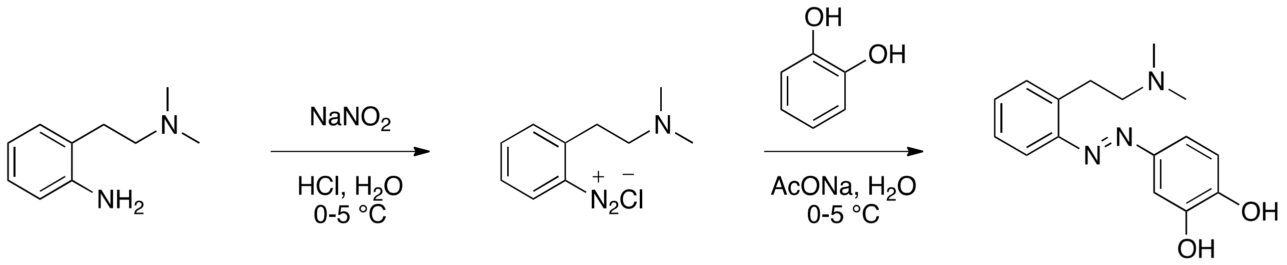

Scheme S1. Chemical synthesis of azodopa.

4-((2-(2-(dimethylamino)ethyl)phenyl)diazenyl)benzene-1,2-diol [azodopa]. In a round-bottom flask, to a mixture of 2-(2-(dimethylamino)ethyl)aniline (165 mg, 1.00 mmol) in water (3 mL) was added concentrated HCl (0.4 mL), and the obtained solution was cooled to 0-5 °C. A precooled solution (0-5 °C) of $NaNO_2$ (76 mg, 1.10 mmol) in water (0.5 mL) was added dropwise to the first solution under vigorous stirring and the resulting mixture was stirred for 30 min at 0-5 °C. The so-obtained yellowish aqueous solution of 2-(2-(dimethylamino)ethyl)benzenediazonium chloride was then added dropwise into a second round-bottom flask containing a mixture of 1,2-dihydroxybenzene (332 mg, 3.00 mmol) and sodium acetate (907 mg, 11.00 mmol) in water (3 mL) at 0-5 °C under vigorous stirring. This final mixture was then allowed to slowly warm up to room temperature and stirred for 12 h (reaction color turned from yellow to dark orange/red). The reaction crude was filtered and then directly purified by reverse-phase HPLC (see the general section above for details) to provide azodopa as a dark orange formate salt (63% yield; purity ≥ 98% as determined by HPLC-PDA analysis).

NMR data are given for the *trans* isomer.

^{1}H NMR (400 MHz, Methanol-d_4) δ 8.54 (s, 1H), 7.65 (dd, J = 7.9, 1.3 Hz, 1H), 7.48 – 7.38 (m, 4H), 7.35 (ddd, J = 7.9, 6.7, 2.1 Hz, 1H), 6.93 (d, J = 8.4 Hz, 1H), 3.51 – 3.40 (m, 2H), 3.15 – 3.05 (m, 2H), 2.71 (s, 6H).

^{13}C NMR (101 MHz, Methanol-d_4) δ 169.95, 151.61, 151.08, 148.20, 147.30, 136.53, 131.92, 131.84, 129.31, 121.15, 116.78, 116.01, 107.52, 60.08, 43.61, 27.83.

R_t (HPLC-PDA) = 6.53 min (*trans*).

HRMS (*m/z*) calculated for $C_{16}H_{20}N_3O_2^+$ [M+H]$^+$: 286.1550, found: 286.1546 (Δ_{ppm} = –1.41).

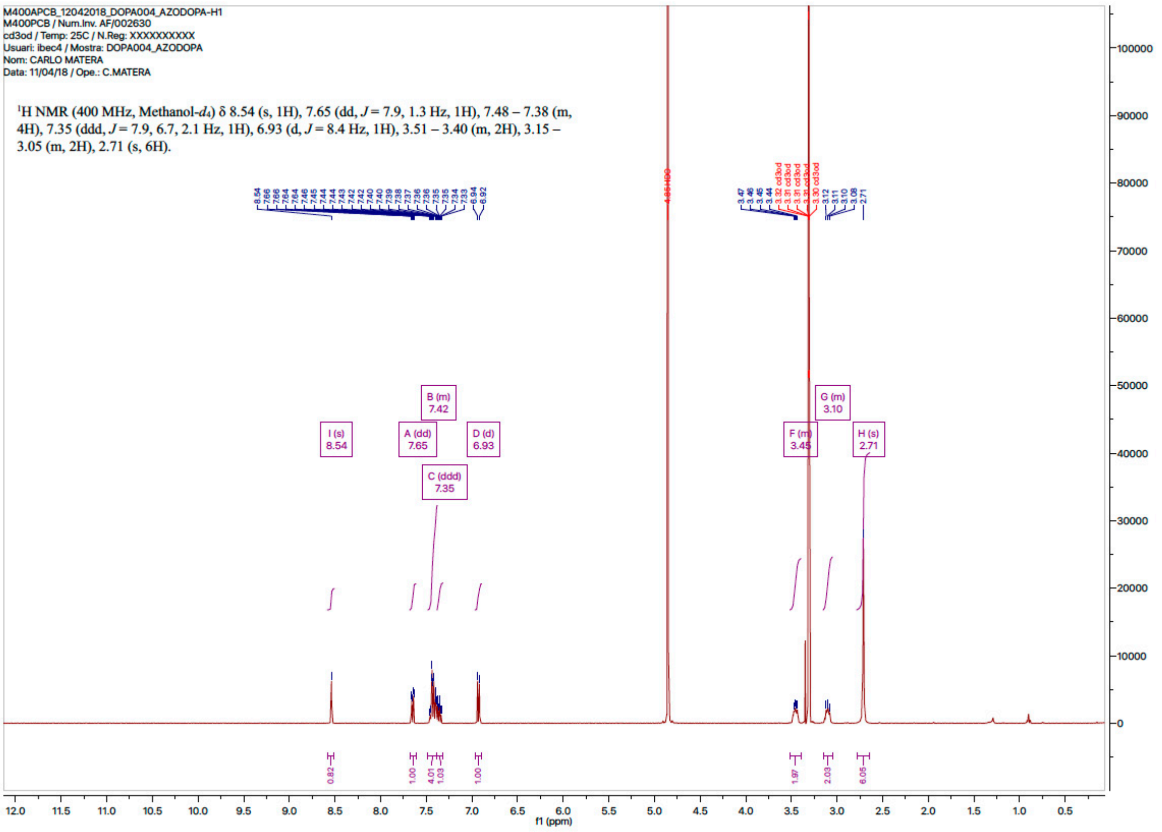

Figure S1. ^{1}H-NMR spectrum of azodopa.

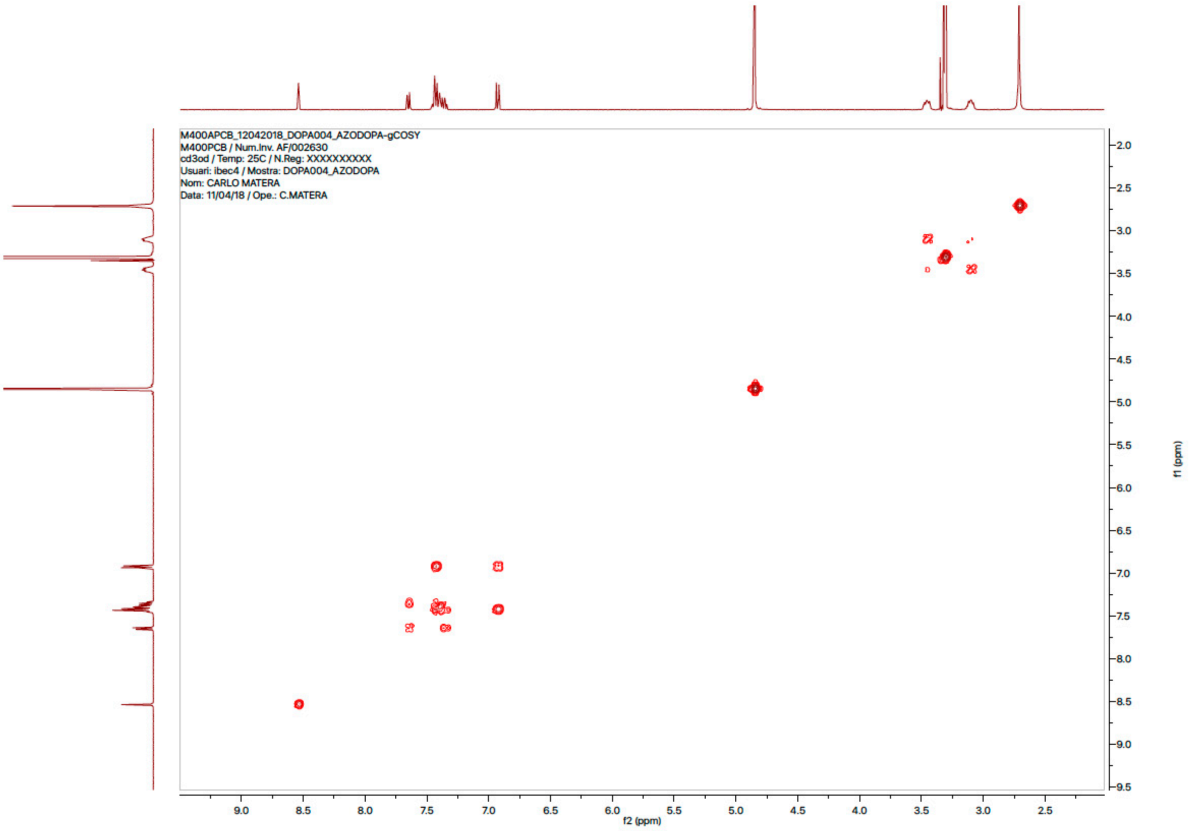

Figure S2. gCOSY spectrum of azodopa.

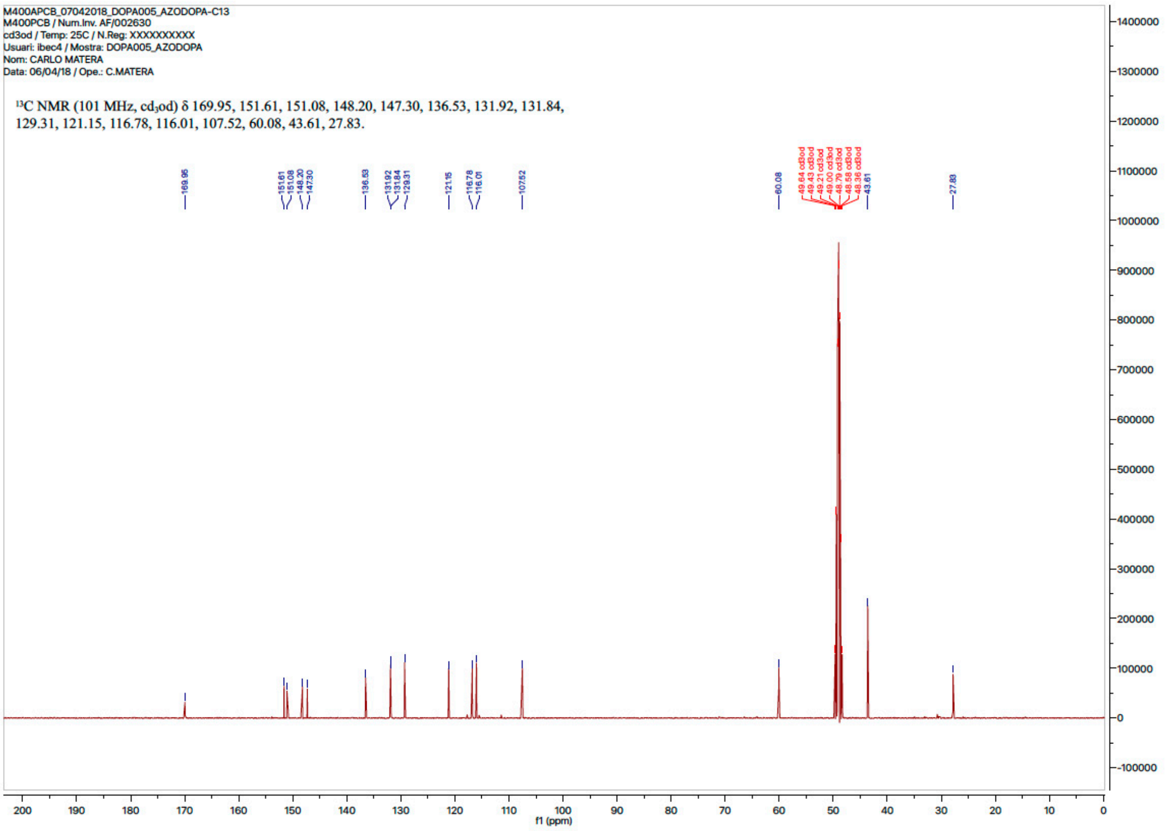

Figure S3. ^{13}C-NMR spectrum of azodopa.

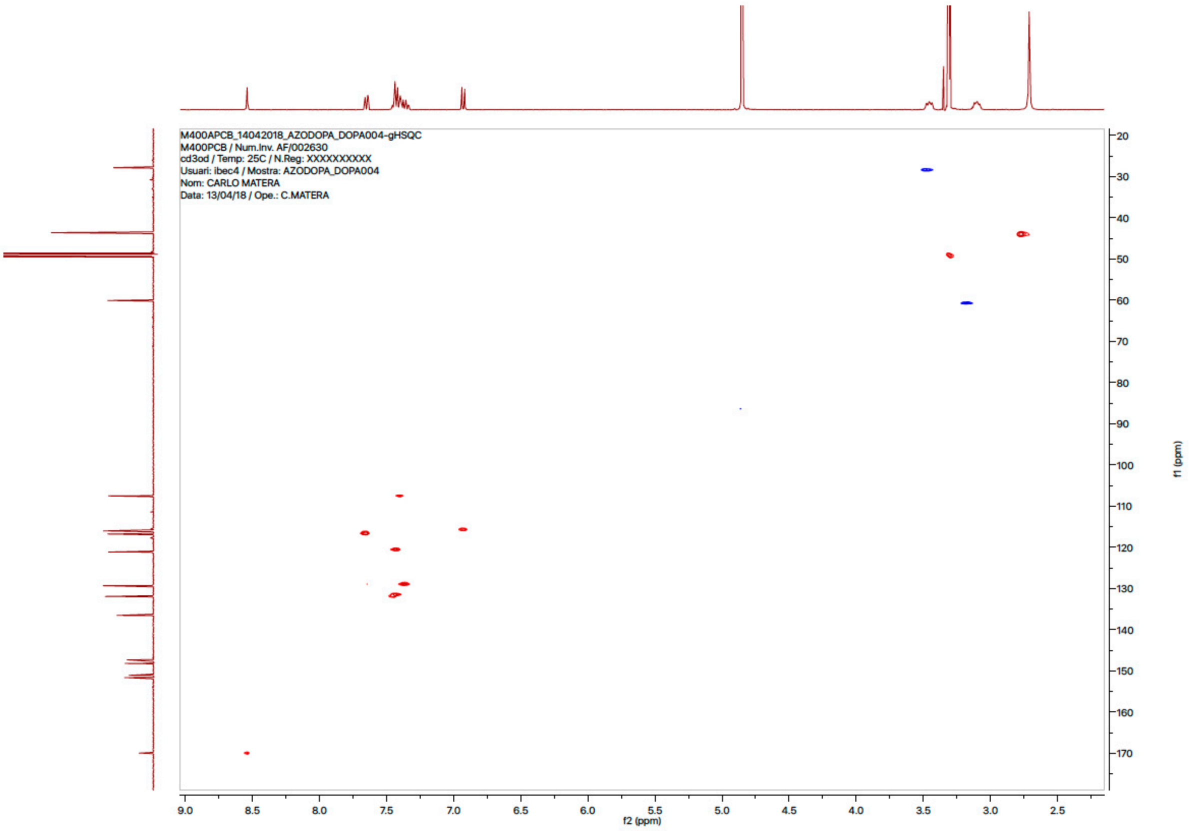

Figure S4. gHSQC NMR spectrum of azodopa.

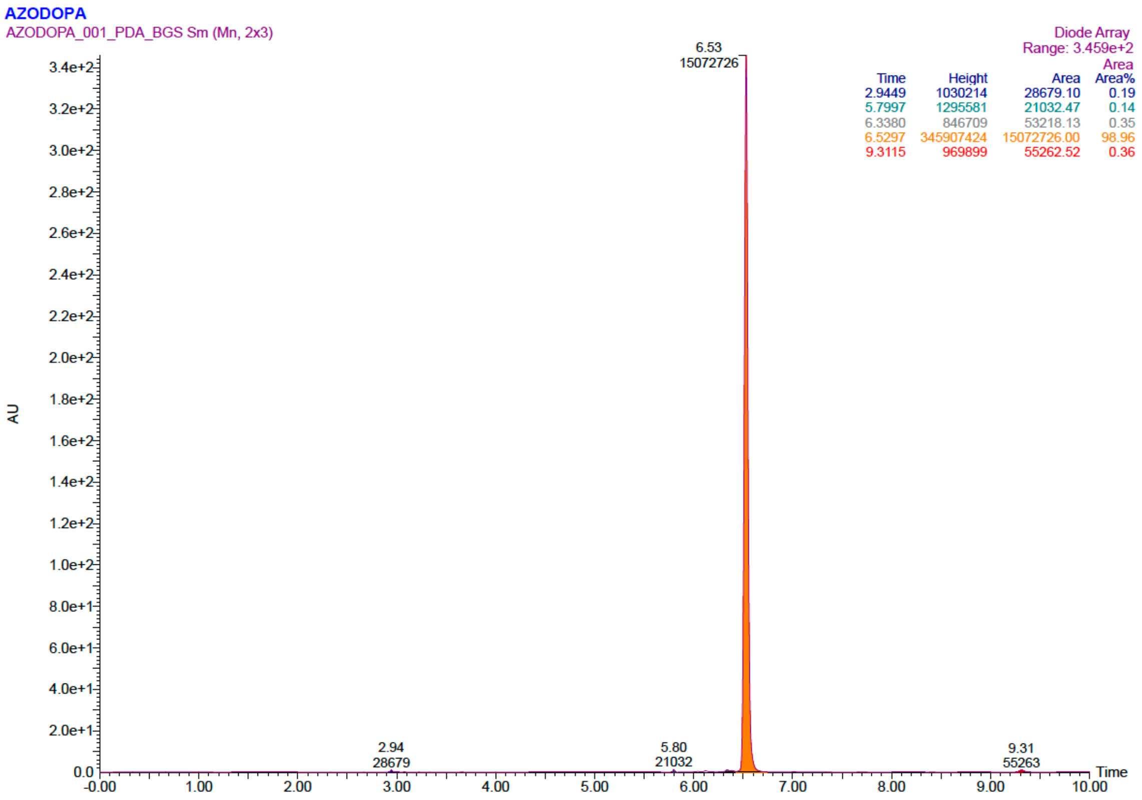

Figure S5. HPLC-PDA chromatogram of azodopa.

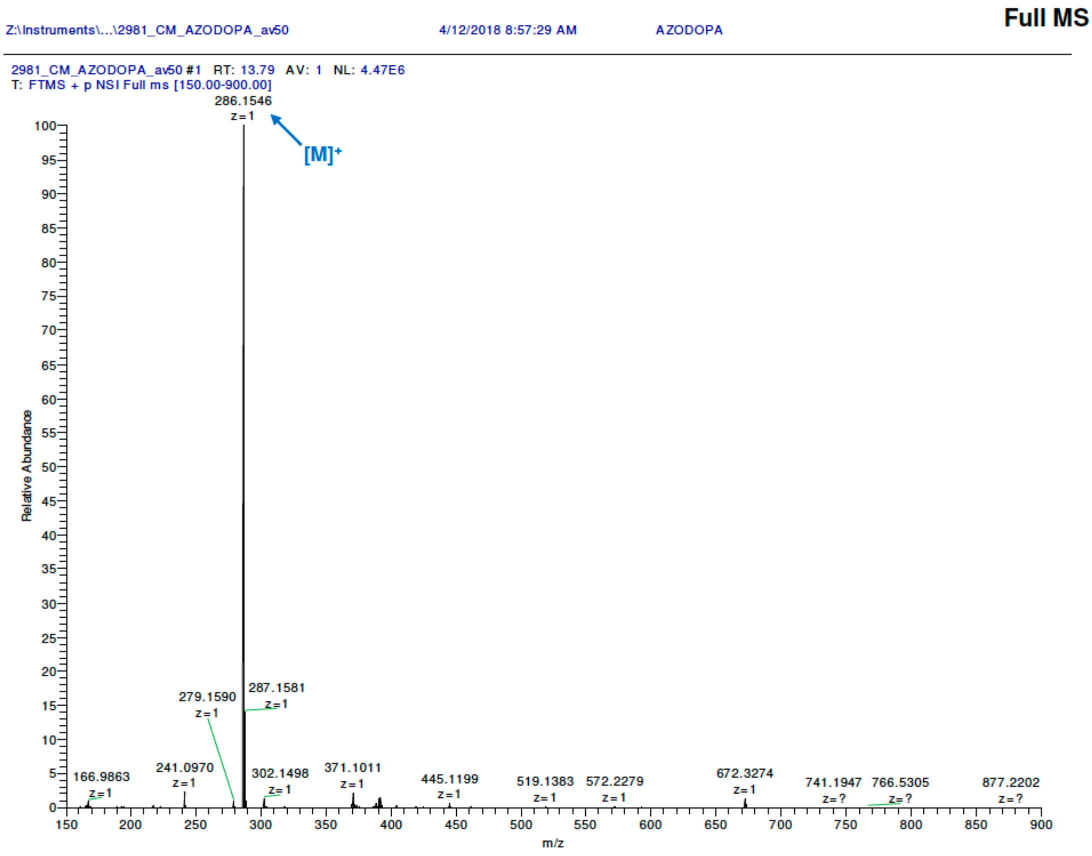

Figure S6. High-resolution mass spectrum of azodopa.

2. Photochemical Characterization

Steady-state UV-Vis spectra were recorded with a Shimadzu UV-1800 UV-VIS Spectrophotometer using standard quartz cuvettes (10 mm optical path). Photoisomerization was accomplished by irradiating azodopa with a Vilber Lourmat UV Lamp (365 nm, 6 W) for 1–3 min. Transient absorption measurements were recorded with a nanosecond laser flash photolysis system (LKII, Applied Photophysics) equipped with a Nd:YAG laser (Brilliant, Quantel) as pump source (λ_{exc} = 355 nm), a Xe lamp as probe source, and a photomultiplier tube (PMT, R928, Hamamatsu) coupled to a spectrograph as detector. Thermal relaxation half-life was estimated applying an exponential one-phase decay model (GraphPad Prism 6).

The thermal relaxation half-life of the *cis* isomer is about 200 µs and thus conversion to the *trans* form occurs almost immediately after turning the light off. For this reason, all biological experiments were performed under continuous illumination.

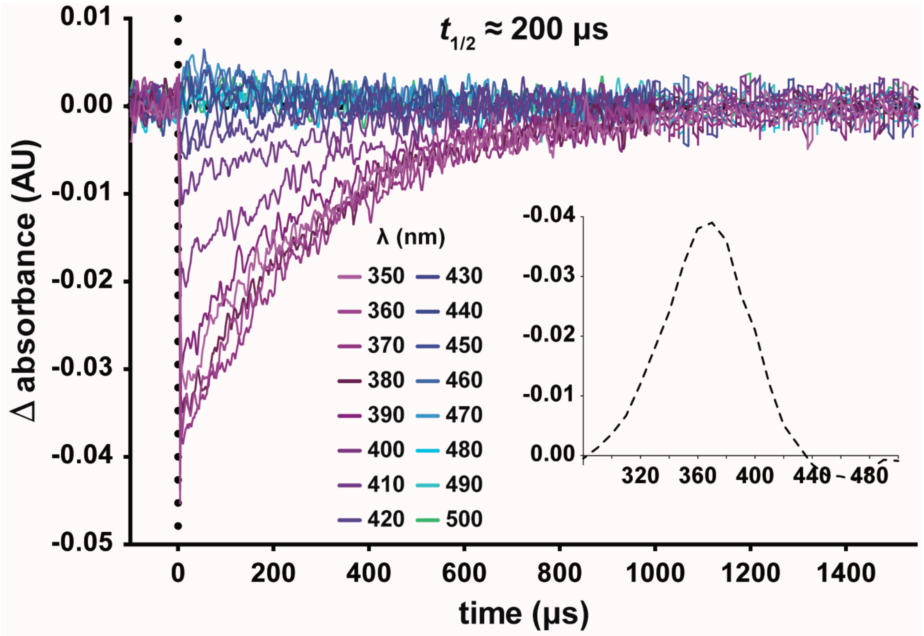

Figure S7. Photochromic behavior of azodopa investigated by transient absorption spectroscopy in water. Transient absorption time traces were measured at different wavelengths upon excitation of *trans*-azodopa (30 µM) with a 5 ns pulsed laser at λ = 355 nm (3 mJ/pulse energy) and 25 °C. Thermal relaxation half-life of the *cis*-isomer (200 µs) was estimated applying an exponential one-phase decay model (GraphPad Prism 6). Inset: Transient absorption spectrum of *trans*-azodopa upon pulsed irradiation at λ = 355 nm recorded at t = 0 µs. X-values represent wavelength (nm), Y-values represent ΔA (arbitrary units, AU).

3. Radioligand Binding Assays

Brains of male and female sheep of 4-6 months old were freshly obtained from the local slaughterhouse. Brain striatum was disrupted with a Polytron homogenizer (PTA 20 TS rotor, setting 3; Kinematica, Basel, Switzerland) for two 5 s periods in 10 volumes of 50 mM Tris-HCl buffer, pH 7.4, containing a proteinase inhibitor cocktail (Sigma, St. Louis, MO, USA). Membranes were obtained by centrifugation twice at 105 000 g for 45 min at 4 °C. The pellet was stored at −80 °C, washed once more as described above and resuspended in 50 mM Tris-HCl buffer for immediate use. Membrane protein was quantified by the bicinchoninic acid method (Pierce Chemical Co., Rockford, IL, USA) using bovine serum albumin dilutions as standard. Binding experiments were performed with membrane suspensions at room temperature in 50 mM Tris-HCl buffer, pH 7.4, containing 10 mM $MgCl_2$.

For D_1-like receptor competition-binding assays, membrane suspensions (0.2 mg of protein/ml) were incubated for 90 min with a constant free concentration of 1 nM (non-irradiated curve) or 3 nM (irradiated curve) of the D_1-like receptor antagonist [^{3}H]SCH23390 (PerkinElmer, Wellesley, MA, USA) and increasing concentrations of azodopa (from 1 nM to 100 µM). For saturation-binding assays, membrane suspensions (0.2 mg of protein/ml) were incubated for 2 h at room temperature in 50 mM Tris-HCl buffer, pH 7.4, containing 10 mM $MgCl_2$ with increasing concentrations of the D_1-like receptor antagonist [^{3}H]SCH23390 (from 0.08 to 7.5 nM of free radioligand concentration for the irradiated curve and from 0.02 to 2.3 nM of free radioligand concentration for the non-irradiated curve) (K_{DA1}(dark): 0.3 ± 0.1* nM, K_{DA1}(UV): 1.6 ± 0.6 nM, (*) $p < 0.05$ vs UV, unpaired *t*-test, n=5). Non-specific binding was determined in the presence of 20 µM of non-labeled SCH23390. Even though both competition curves appear similar, due to the lower affinity of the radioligand upon illumination (Figure S8a), we calculated an almost 4-fold decrease in affinity at 365 nm (see Table S1).

For D_2-like receptor competition-binding assays, membrane suspensions (0.2 mg of protein/ml) were incubated for 2 h with a constant free concentration of 0.8 nM of the D_2 antagonist [^{3}H]YM-09151-2 (K_{DA1} = 0.30 nM) and increasing concentrations of azodopa. Nonspecific binding was determined in the presence of 30 µM of dopamine because at this concentration dopamine does not displace the radioligand from sigma receptors.

During the incubation period, incubates were irradiated or not with UV light. Experiments under illumination were performed by continuously irradiating with a Vilber Lourmat transilluminator (model ECX-F20.L, 365 nm, 6 x 8 W). In both binding assays, free and membrane-bound ligands were separated by rapid filtration of 500 µl aliquots in a cell harvester (Brandel, Gaithersburg, MD, USA) through Whatman GF/C filters embedded in 0.3% polyethylenimine that were subsequently washed for 5 s with 5 ml of ice-cold 50 mM Tris-HCl buffer. The filters were incubated with 10 mL of Ultima Gold MV scintillation cocktail (PerkinElmer) overnight at room temperature and radioactivity counts were determined using a Tri-Carb 2800 TR scintillation counter (PerkinElmer) with an efficiency of 62%.

Data were analyzed according to the “dimer model” developed by Casadó and collaborators.[1] The model assumes GPCR dimers as a main functional unit and provides a more robust analysis of parameters obtained from saturation and competition experiments with orthosteric ligands, as compared with the commonly used “two-independent-site model”.[1,2] The equation describing the saturation experiment in non-cooperative conditions is:

$$A_{bound} = \frac{2\,A\,R_T}{2K_{DA1} + A}$$

where A represents the radioligand concentration, R_T the total number of dimers (being $B_{max} = 2R_T$), and K_{DA} the affinity of the radioligand.

To calculate the macroscopic equilibrium dissociation constants from competition experiments, when A and the competitor B are both non-cooperative and there is non-allosteric modulation between A and B, the following equation was applied:

$$A_{bound} = \frac{\left(4\,K_{DA1}\,A + 2\,A^2 + \frac{2\,K_{DA1}\,A\,B}{K_{DB1}}\right) R_T}{4\,K_{DA1}^2 + 4\,K_{DA1}\,A + A^2 + \frac{2\,K_{DA1}\,A\,B}{K_{DB1}} + \frac{4\,K_{DA1}^2\,B}{K_{DB1}} + \frac{K_{DA1}^2\,B^2}{K_{DB1}^2}}$$

where B represents the assayed competing compound concentration and K_{DB} the affinity of the competing ligand.

Radioligand competition and saturation curves were analyzed by nonlinear regression using the commercial Grafit curve-fitting software (Erithacus Software, Surrey, UK), by fitting the binding data to the mechanistic dimer receptor model, as described in detail elsewhere.[3]

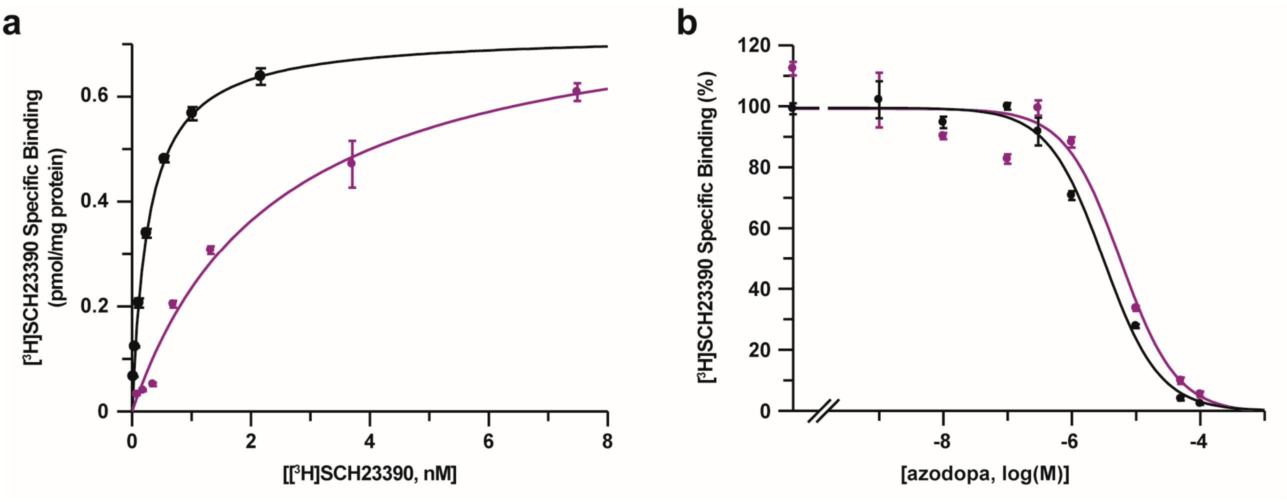

Figure S8. (a) Radioligand saturation binding experiments. Saturation experiments for D_1 in sheep brain striatum membranes. Saturation assays (0.2 mg protein/ml) were performed, and data were fitted as indicated above. These experiments were performed with increasing concentrations of the D_1 antagonist [^{3}H]SCH23390 in the presence (purple) or absence (black) of UV irradiation during the incubation period of 1 h. Data are mean ± S.E.M. values from a representative experiment performed in triplicate. Affinity values of [^{3}H]SCH23390 appear in Table S1. **(b) Radioligand competition binding experiments.** Competition experiments for D_1 in sheep brain striatum membranes. Competition assays (0.2 mg protein/ml) were performed, and data were fitted as indicated above. The D_1 antagonist [^{3}H]SCH23390 at 1 nM was displaced with increasing concentrations of azodopa in the presence (purple) or absence (black) of UV irradiation during the incubation period. Data are mean ± S.E.M. values from a representative experiment performed in triplicate. 100% of [^{3}H]SCH23390 specific binding = 1.1 ± 0.1 pmol/mg protein (n = 9 experiments performed in triplicate). Affinity values of azodopa (K_{DB}) appear in Table S1.

	D_1 (*)	D_2 (*)
**dark**	**K_{DB}: 600 ± 100 nM**	**K_{DB}: 210 ± 30 nM**
**UV**	**K_{DB}: 2200 ± 600 nM**	**K_{DB}: 900 ± 300 nM**

Table S1. Binding affinity of azodopa for D_1 and D_2 in the dark and under illumination. Affinity values obtained by fitting data from competition experiments of [^{3}H]SCH23390 and [^{3}H]YM-09151-2 *vs* azodopa to the dimer receptor model. Data are mean ± S.E.M. from 3–5 experiments performed with different striatal homogenates of 3–4 animals. Statistical differences between the values obtained were calculated by an unpaired *t*-test. (*) $p < 0.05$ for affinity under UV vs in dark.

In conclusion, we found that azodopa has 3-fold greater affinity for D_2-like than D_1-like receptors, and in both cases the affinity is higher in the dark (*trans* isomer).

4. cAMP Accumulation Assays

Cell culture and transfection. For cAMP determinations, the human receptor construct of D_1 receptor fused to full-length yellow variant of green fluorescent protein (EYFP) was used.[4] 2 µg of plasmid cDNA was transfected into HEK-293T cells using polyethylenimine (Sigma-Aldrich) in 25-cm^2 cell culture flasks. Cells were maintained in culture with Dulbecco's modified Eagle's medium (Sigma-Aldrich) supplemented with 2

mM L-glutamine, MEM nonessential amino acid solution (1/100), 100U/ml penicillin/streptomycin, and 5% (vol/vol) of heat-inactivated fetal bovine serum and kept in an incubator at 37 °C and 5% CO_2. All experiments were performed approximately 48 hours after transfection.

Experimental protocol. Homogeneous time-resolved fluorescence energy transfer (HTRF) assays were performed using the Lance Ultra cAMP kit (PerkinElmer), based on competitive displacement of a europium chelate-labelled cAMP tracer bound to a specific antibody conjugated to acceptor beads. We first established the optimal cell density which provides a response that covers most of the dynamic range of the cAMP standard curve. Thus, 1200 cells in growing in medium containing 30 µM zardaverine were put into each well of a white ProxiPlate 384-well microplate (PerkinElmer). Cells were pretreated or not with the D_1-like receptor antagonists SKF83566 (Tocris) for 5 min before the activation with azodopa for 15 min. During these 15 minutes, cells were irradiated or not with UV light to induce the *trans*/*cis* isomerization of the compound. Experiments under illumination were performed by continuously irradiating with a Vilber Lourmat transilluminator (model ECX-F20.L, 365 nm, 6 x 8 W). Fluorescence at 665 nm was analyzed on a PHERAstar Flagship microplate reader equipped with an HTRF optical module (BMGLab technologies, Offenburg, Germany). cAMP assays were also performed in non-transfected HEK-293T cells. In these cells, the effect of azodopa at 10 µM, dopamine at 1 µM and the D_1-like receptor agonist SKF38393 at 300 nM was tested to ensure that all the effects observed were D_1-dependent. Statistical differences were analyzed by two-way ANOVA followed by Tukey's post-hoc test (GraphPad Prism 6).

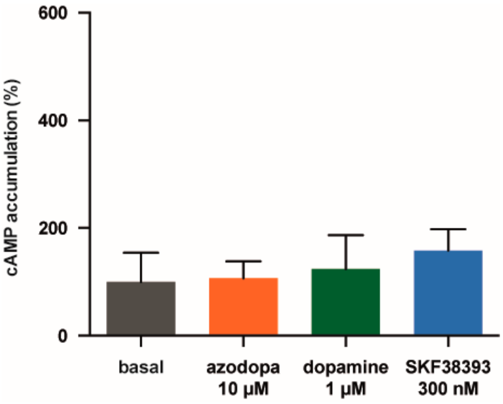

Figure S9. Effect of D1 agonists on the adenylyl cyclase activity in non-transfected HEK-293T cells. As a negative control experiment, cAMP accumulation was determined in non-transfected HEK-293T cells activated with the D_1 dopamine agonists azodopa (10 µM, orange), dopamine (1 µM, green) or SKF38393 (300 nM, blue) for 15 minutes. Values are represented in % vs basal levels (gray) of cAMP. Data are mean ± S.E.M. of 3 experiments performed in quadruplicate.

5. ERK Phosphorylation Assays

Cell culture and transfection. For ERK1/2 phosphorylation determinations, the human receptor construct of D_1 receptor fused to full-length yellow variant of green fluorescent protein (EYFP) was used.[4] 2 µg of plasmid cDNA was transfected into HEK-293T cells using polyethylenimine (Sigma-Aldrich) in 25 cm^2 cell culture flasks. Cells were maintained in culture with Dulbecco's modified Eagle's medium (Sigma-Aldrich) supplemented with 2 mM L-glutamine, MEM nonessential amino acid solution (1/100), 100U/ml penicillin/streptomycin, and 5% (vol/vol) of heat-inactivated fetal bovine serum and kept in an incubator at 37 °C and 5% CO_2. All experiments were performed approximately 48 hours after transfection.

Experimental protocol. The day of the experiment, cells were starved by treating them with serum free media for 3-4 h at 37 °C. After that, cells were incubated with the indicated antagonist for 5 min and then, with azodopa for 7 min at 37 °C. As for cAMP assays, cells were irradiated or not with UV light during the azodopa incubation. Experiments under illumination were performed by continuously irradiating with a Vilber Lourmat transilluminator (model ECX-F20.L, 365 nm, 6 x 8 W). Then, cells were rinsed with ice-cold

phosphate-buffered saline and lysed by adding 200 ml ice-cold lysis buffer (50 mM Tris-HCl [pH 7.4], 50 mM NaF, 150 mM NaCl, 45 mM β-glycerophosphate, 1% Triton X-100, 20 mM phenyl-arsine oxide, 0.4 mM $NaVO_4$, and protease inhibitor cocktail). The cellular debris was removed by centrifugation at 13 000 g for 5 min at 4 °C, and the protein was quantified. To determine the level of ERK1/2 phosphorylation, equivalent amounts of protein were separated by electrophoresis on a denaturing 10% SDS polyacrylamide gel and transferred onto polyvinylidene difluoride membranes. Odyssey blocking buffer (LI-COR Biosciences, Lincoln, NE) was then added, and the membrane was rocked for 90 minutes. The membranes were then probed with a mixture of a mouse anti–phospho-ERK1/2 antibody (1:2500; Sigma-Aldrich) and rabbit anti-ERK1/2 antibody that recognizes both phosphorylated and nonphosphorylated ERK1/2 (1:40000; Sigma-Aldrich) overnight at 4 °C. The 42- and 44-kDa bands corresponding to ERK1 and ERK2 were visualized by the addition of a mixture of IRDye 800 (anti-mouse) antibody (1:10000; Sigma-Aldrich) and IRDye 680 (anti-rabbit) antibody (1:10000; Sigma-Aldrich) for 2 hours and scanned by the Odyssey infrared scanner (LICOR Biosciences). Band densities were quantified using the scanner software and exported to Excel (Microsoft, Redmond, WA). The level of phosphorylated ERK1/2 isoforms was normalized for differences in loading using the total ERK1/2 protein band intensities. Statistical differences were analyzed by two-way ANOVA followed by Tukey's post-hoc test (GraphPad Prism 6).

6. Ca^{2+} Imaging Assays

Cell culture and transfection. HEK-293T cells were purchased from the European Collection of Authenticated Cell Culture. Cells were maintained at 37 °C in a humidified atmosphere with 5% CO_2 and grown in Dulbecco's Modified Eagle's Medium (DMEM) and Ham's F-12 Nutrient Mixture (DMEM/F12 1:1, Life Technologies), supplemented with 10% fetal bovine serum (FBS, Life Technologies) and antibiotics (1% penicillin/streptomycin, Sigma-Aldrich). Plasmid pcDNA 3.1 (+) encoding human D_1 receptor was obtained as a kind gift from Ewa Błasiak (Department of Physical Biochemistry, Jagiellonian University, Kraków, Poland). Transient expression of the human D_1 dopamine receptor and the genetically encoded calcium indicator RGECO-1 (Addgene) (ratio1:1) was induced by using the X-tremeGENE 9 DNA Transfection Reagent (Roche Applied Science) following the manufacturer's instructions. The day after, cells were harvested with Accutase® (Sigma-Aldrich) and seeded onto 16 mm glass coverslips (Fisher Scientific) pretreated with poly-L-Lysine (Sigma-Aldrich) to favor cell adhesion. Preconfluent cultures were used for experiments at 48–72 hours after transfection.

Experimental protocol. The bath solution used for single cell intracellular calcium recordings contained: 140 mM NaCl, 5.4 mM KCl, 1 mM $MgCl_2$, 10 mM HEPES, 10 mM glucose and 2 mM $CaCl_2$, and was adjusted to pH 7.40 with aqueous NaOH. Before each experiment, cells were mounted on the recording chamber (Open Diamond Bath Imaging Chamber for Round Coverslips from Warner Instruments). Cells were rinsed with fresh solution, and the recording chamber was filled with 1 ml recording solution and placed on an IX71 inverted microscope (Olympus) with a XLUMPLFLN 20XW x20/1 water immersion objective (Olympus). R-GECO1 was excited during 50 ms at 562 nm by using a Polychrome V light source (Till Photonics) equipped with a Xenon Short Arc lamp (Ushio) and a 585 nm dichroic beam splitter (Chroma Technology). Emission at 600 nm was filtered by ET630/75nm emission filter (Chroma Technology) and finally collected by a C9100-13 EM-CCD camera (HAMAMATSU). Images were acquired at room temperature with an imaging interval of 4 s with the SmartLux software (HEKA). Imaging analysis was performed with FIJI (ImageJ). Dopamine (50 μM, Sigma-Aldrich) was used as agonist to stimulate D_1 receptors expressed in HEK-293T cells. Addition of dopamine, azodopa or vehicle (0.1% DMSO) was carried out by carefully pipetting a small volume during image acquisition into the accessory pool of the recording chamber to assure a good mixing of the solution. Photoisomerization of azodopa was achieved by continuously irradiating with a Vilber Lourmat UV Lamp (365 nm, 6 W) for 3 minutes before application. R-GECO1 is a red-shifted Ca^{2+} fluorescent indicator and it was chosen because imaging at longer wavelength minimizes the effect of the unwanted fluorescence generated by continuously illuminating the specimen with 365 nm light. Data were normalized over the maximum response obtained with dopamine at 50 μM. The Origin 8 software was used to calculate the values of the

peak amplitude and the values of the area under the curve (AUC). AUC values correspond to the integral of the curves over each drug application interval. Statistical differences were analyzed by one-way ANOVA followed by Tukey's post-hoc test (GraphPad Prism 6).

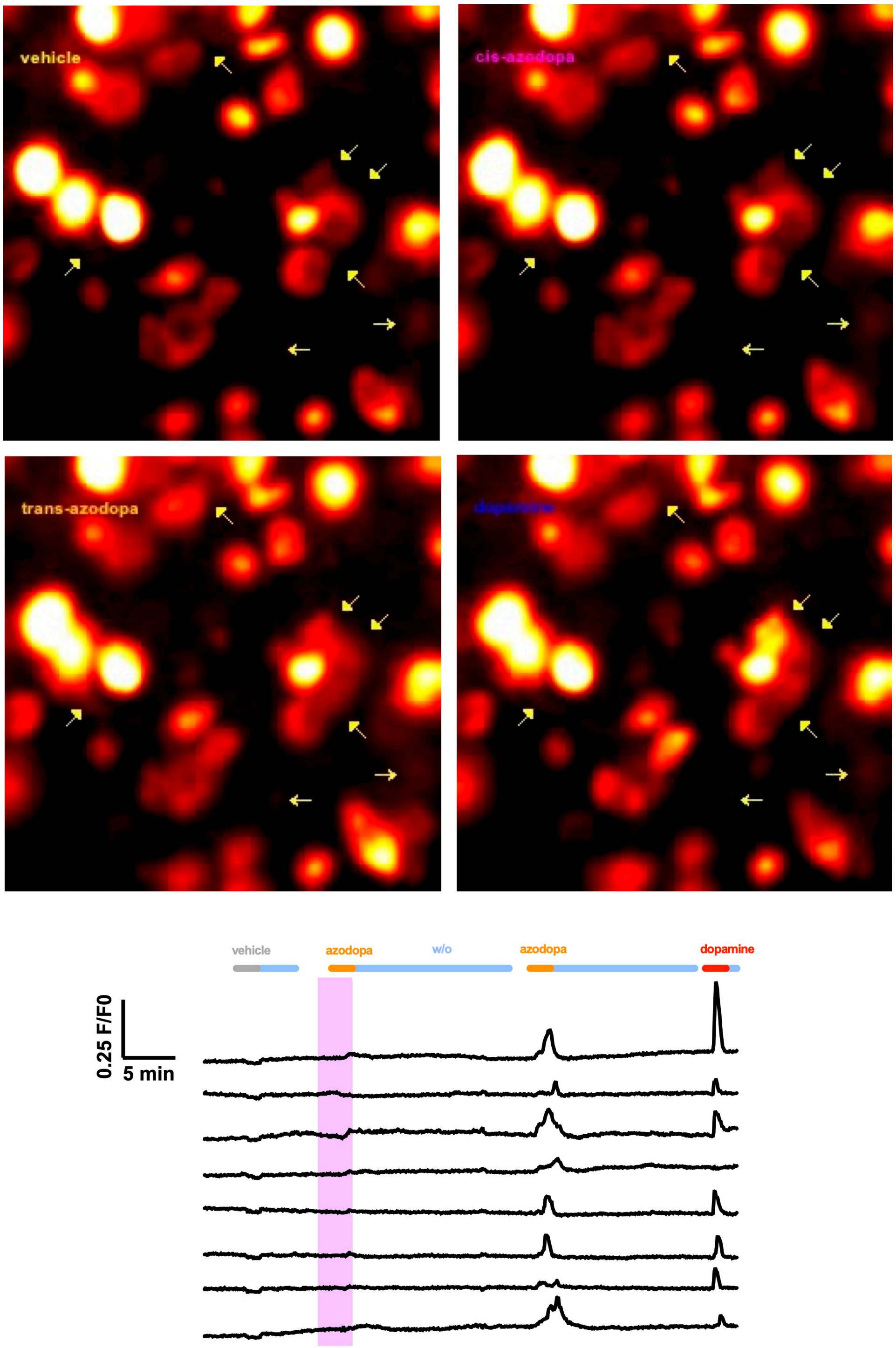

Figure S10. Effect of azodopa on D_1-mediated intracellular calcium release. Example frames from Supplementary Movie 2 (top images; arrows indicating cells responding to *trans*-azodopa and dopamine over the time course of the experiment) and real-time calcium imaging response traces (bottom panel) in HEK-293T cells co-expressing D_1 receptors and R-GECO1 as calcium indicator. Single-cell calcium traces for 8 representative cells are shown. Traces were recorded upon direct application of azodopa (50 µM, orange bars) in the dark (white area) and under illumination (purple area). Shadow represents '± S.E.M.'. Gray and green bars indicate the application of vehicle (control) and dopamine (reference agonist), respectively. Light blue bars indicate wash-out periods.

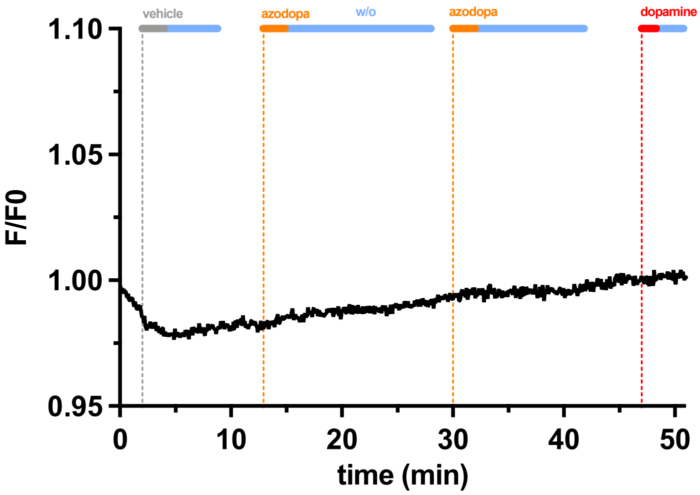

Figure S11. Calcium imaging experiments in control cells. Real-time calcium imaging in HEK-293T cells (n = 25) expressing R-GECO1 but not D_1. No calcium oscillations were recorded upon the application of vehicle (gray), azodopa (orange) under illumination and in the dark, or dopamine (green). Data are mean ± S.E.M.

7. Behavioral Assays in Zebrafish

Animal housing. Wild-type zebrafish embryos (Tupfel long-fin strain) were purchased from the animal facility of the Barcelona Biomedical Research Park (PRBB) and raised in darkness for 6 days at 28.5 °C in UV filtered tap water in Petri dishes (daily cleaned and refilled). Animal development was checked every 24 hours. Unhealthy or abnormal embryos and larvae were removed and euthanatized in tricaine methanesulfonate 0.02%. All experiments and procedures were conducted according to the European Directive 2010/63/EU.

Blindness induction. For the experiments with blinded zebrafish, larvae underwent a non-invasive and highly specific blinding technique that induces photoreceptor apoptosis in the dorsal and central retina.[5] The light-lesioning procedure was performed in a thermostatic and well-ventilated room equipped with small fans for air circulation and heat dissipation. Larvae at 5 days post-fertilization (dpf) were placed inside a 25 mm Petri dish containing 20 ml of UV filtered tap water, inserted in a closed mirrored chamber, and then exposed to 135 000 lux light emitted by a mercury lamp (model Olympus U-LH100HG) for 30 min. Temperature was monitored throughout the whole procedure.

Assay protocol. Behavioral studies were conducted on wild-type zebrafish larvae at 6 dpf. Vehicle and compounds were added with a multichannel pipette to exclude differences related to a delay in the application of each solution. Movements were recorded and analyzed using the ZebraBox tracking system and the ZebraLab software (ViewPoint Life Science, France). On the morning of the test, 6 dpf larvae were moved into a new batch of fresh water and checked for motility capabilities and possible physical mutations. Larvae were then randomly divided into control and treatment groups. Each individual was placed in a separate well of a 96 well plate, each containing 200 µl of fresh UV filtered water and left undisturbed in the dark for 30 min to get acquainted with the new setting (habituation time). Afterwards, 100 µl of water were removed from each well and replaced with 100 µl of a double concentrated vehicle or treatment solution. At this point, the plate was inserted into the ZebraBox and the recording period started. Activity was recorded for a total of 62.5 min. Animals were exposed to controlled cycles of dark and 365 nm UV light, using the following protocol of illumination: dark (20 min, for adaptation), UV light (30 sec), dark (20 min), and then four cycles of UV light (30 sec) and dark (5 min). Illumination at 365 nm was performed with a built-in array of 12 LEDs placed 12 cm away from the multiwell plate. Light intensity, measured with a Newport 1916-C optical power meter coupled to a Newport 918D-SL-OD3R detector, was 5.9 $mW \cdot cm^{-2}$. All experiments were conducted at 12.00 pm (UTC+01:00).

Tracking and analysis of swimming activity. Alterations of locomotor activity were determined by monitoring and measuring fast movements, swimming distances and duration of high-speed swimming. In particular, the dependent variables measured included: total distance travelled, distance travelled at ≤ 2 mm·s^{-1}, distance travelled at 2–6 mm·s^{-1}, distance travelled at ≥ 6 mm·s^{-1}, time spent moving, time spent freezing, and number of bursts. The arbitrary cut-off for motility (6 mm·s^{-1}) was chosen because no variance was found among the groups at lower speeds. Total movements were not considered to limit the error of the video recording on minimal movements. Data were analyzed by two-way ANOVA with Tukey's post-hoc test or uncorrected Fisher's least significant difference test for statistical significance (GraphPad Prism 6).

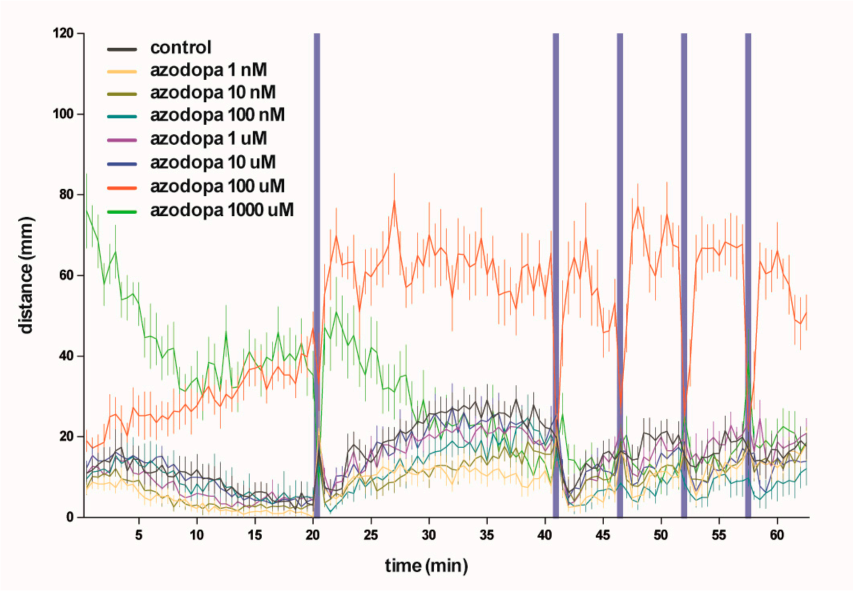

Figure S12. Effects of azodopa on the locomotor activity of zebrafish larvae. Swimming activity (distance moved over time) in larvae exposed to vehicle (control group) or azodopa in a wide range of concentrations (from 1 nM to 1 mM) in the dark (white areas) or under illumination with 365 nm light (purple bars). Up to a concentration of 10 µM azodopa, no significant differences in the locomotor activity were detected in comparison with the control group. A great increase of the swimming activity was recorded at 1 mM (green line), but the effect eventually disappeared in about 30 min, when fish were possibly exhausted. The most interesting and representative alterations of the behavioral profile were observed at 100 µM. Only fast movements (speed ≥ 6 mm·s^{-1}) were considered and integrated every 30 s. Data are mean ± S.E.M. (n = 11–12 individuals/condition).

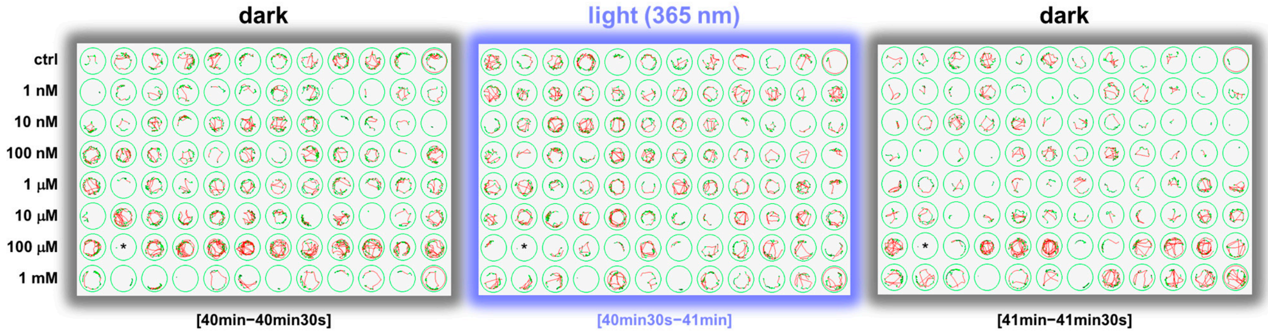

Figure S13. Tracking of zebrafish larvae movement in a 96-well plate. Trajectories of individual larvae treated with the vehicle (control group) or azodopa (from 1 nM to 1 mM) in different lighting conditions. Green lines and red lines indicate slow and fast swimming periods, respectively. Larvae treated with 100 µM azodopa show higher activity in the dark in comparison with the controls, while they returned to control levels of activity under illumination. Trajectories were extrapolated from the same experiment represented in Figure 3 (panels a–d) and Figure S12. (*) Dead larvae excluded from the analysis.

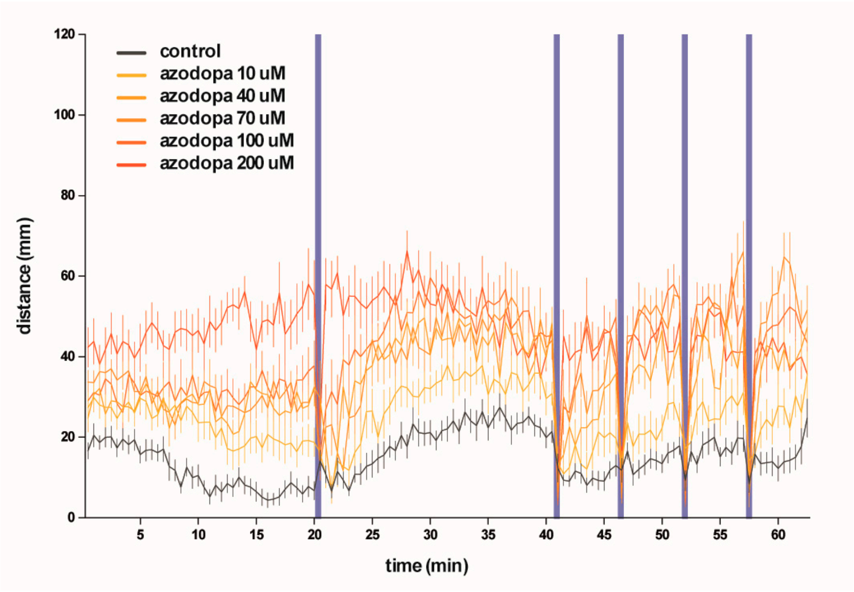

Figure S14. Effects of azodopa on the locomotor activity of zebrafish larvae. Swimming activity (distance moved over time) in larvae exposed to vehicle (control group) or azodopa in a small range of concentrations (from 10 µM to 200 µM) in the dark (white areas) or under illumination with 365 nm light (purple bars). Azodopa induced a dose-dependent increase of the swimming activity in the dark. Only fast movements (speed ≥ 6 mm·s^{-1}) were considered and integrated every 30 s. Data are mean ± S.E.M. (n = 12 individuals/condition).

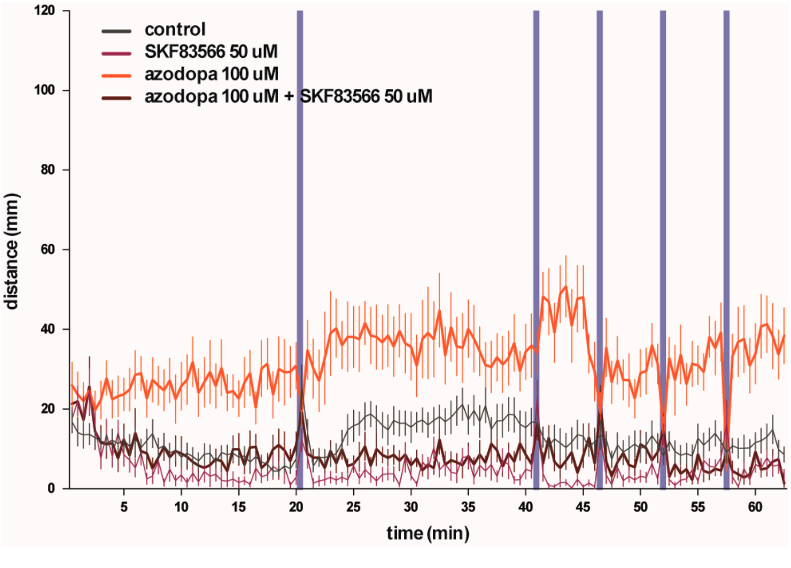

Figure S15. The effects of azodopa on zebrafish locomotor activity are counteracted by a D$_1$ antagonist. (a) Swimming activity (distance moved over time) in larvae exposed to vehicle (control group), azodopa (100 µM), SKF83566 (50 µM, D$_1$-selective antagonist), or azodopa + SKF83566 (100 µM and 50 µM, respectively), in the dark (white areas) or under illumination with 365 nm light (purple bars). The co-application of a D$_1$ antagonist abolished the behavioral effects produced by azodopa, restoring a more basal level of activity (brown line), and suggesting that azodopa locomotor effects are D$_1$-mediated. Only fast movements (speed ≥ 6 mm·s^{-1}) were considered and integrated every 30 s. Data are mean ± S.E.M. (n = 12 individuals/condition).

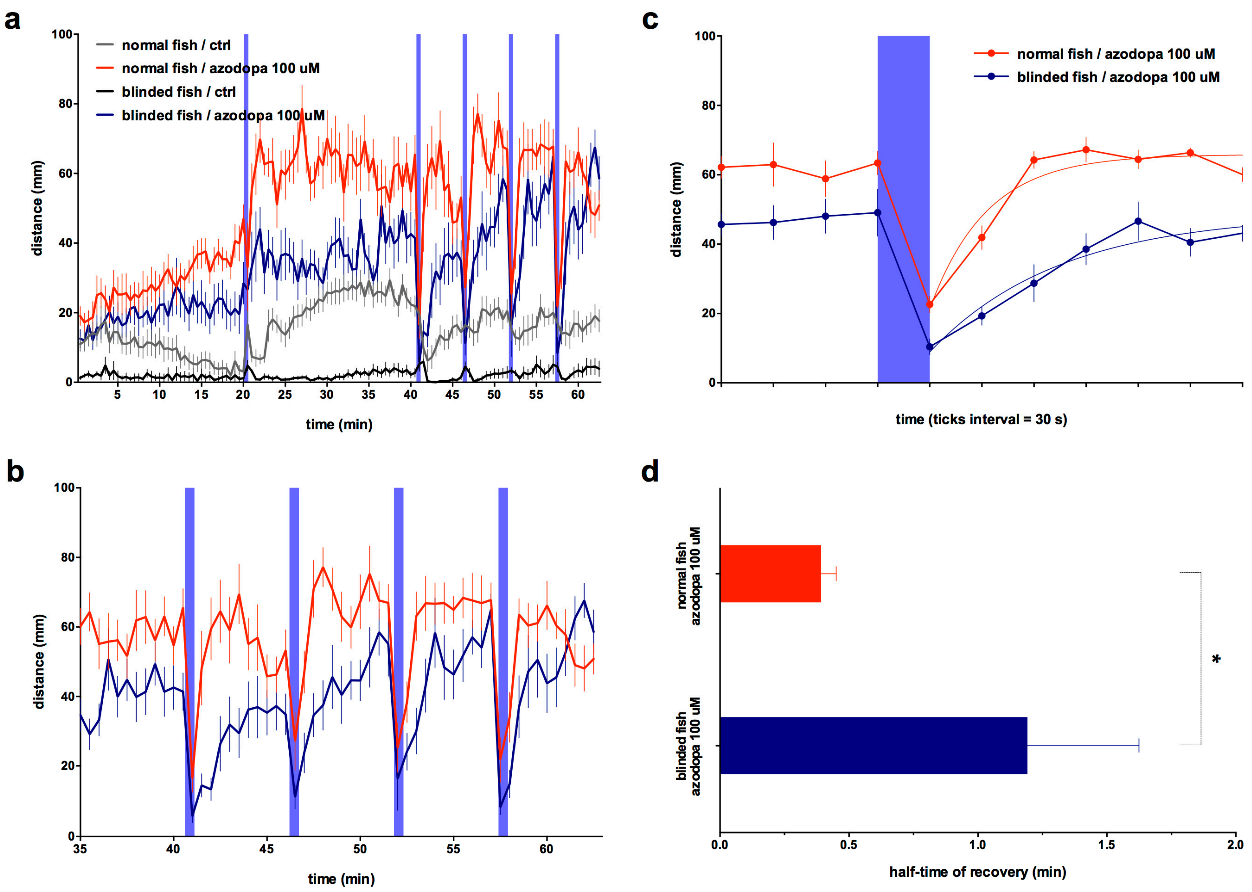

Figure S16. Azodopa produces faster behavioral responses in normal fish than in blinded fish upon *cis*-to-*trans* isomerization. (a) Swimming activity (distance moved over time) in normal and blinded zebrafish larvae exposed to vehicle (control group) or azodopa (100 µM) in the dark (white areas) or under illumination with 365 nm light (purple bars). (b) Zoomed view of the last 4 dark-light cycles from panel 'a' to compare the kinetics of photoresponse in normal fish versus blinded fish. (c) Average of swimming activity (distance moved over time) in normal and blinded zebrafish larvae exposed to azodopa (n = 11-12 individuals/condition, 4 last consecutive dark-light-dark periods). Blinded zebrafish needed more time to recover the maximum level of activity when the light was switched off, likely because of the absence of visual response. The recovery of activity in normal and blinded fish fitted a pseudo-first-order model (non-linear regression based on one-phase association kinetics; $R^2_{normal} = 0.8553$, $R^2_{blinded} = 0.7168$; solid curves in orange and blue), with statistically different rate constants ($K_{normal} = 1.83 \pm 0.39$, $K_{blinded} = 0.80 \pm 0.35$; F test, $p < 0.05$). (d) In order to statistically compare the half-times of recovery, data points from each dark-light-dark period were analyzed independently by nonlinear regression and refitted to a one-phase association curve. The four half-times from either the normal group or the blinded group curves were then averaged and analyzed by an unpaired two-tailed *t*-test (* $p < 0.05$). All analyses were performed with GraphPad Prism 6.

8. Electrophysiological Recordings in Mice

Animals. Young adult male mice (C57BL/6, n = 4) were obtained from the local colony at the Barcelona Biomedical Research Park Animal Facility. Mice were 3 months old and weighed 20-25 g at the time of the experiments. All procedures were conducted in compliance with EU directive 2010/63/EU and Spanish guidelines (Laws 32/2007, 6/2013 and Real Decreto 53/2013) and were authorized by the local Animal Research Ethics Committee and the local government (Generalitat de Catalunya).

Surgeries. Mice were anesthetized with isoflurane at 0.5-2% and placed in a stereotaxic apparatus. A heating pad was placed between the animal and the stereotaxic frame to maintain body temperature. The level of anesthesia was regularly tested by tail-pinching, retraction of the hind paws and changes in body temperature. A craniotomy was unilaterally drilled above the secondary motor cortex (M2: AP +2.0 mm, L

0.8 mm from bregma). Dental cement was used to build a small pool around the craniotomy that contained the saline or azodopa solutions. One custom-made octrode with 8 independent electrodes (4 two-wire stereotrodes) was inserted approximately 200 μm deep into the superficial layers of the M2. The stereotrodes were made by twisting a strand of tungsten wire of 25 μm of diameter (Advent, UK) and had impedances that ranged from 100 to 400 kΩ.

Electrophysiological recordings and pharmacology. Neural signals were recorded with the multi-channel Open Ephys system at a sampling rate of 30 kHz. The electrode wires were pinned to an adaptor to facilitate their connection to an Intan RHD2132 preamplifier that bandpass filtered (0.1-6 kHz) and digitized the analogic signals. These were later amplified and processed by the Open Ephys data acquisition system and finally visualized and stored in a PC via the Open Ephys GUI software. During the recordings, the exposed electrophysiological parts (octrode, adaptor, amplifier, LEDs) were shielded with aluminum foil to prevent environmental and electrical noise to interfere with the recordings. Experiments started when the anesthesia level and the electrophysiological signals were stable for 10 minutes. We recorded neural signals for 10 minutes under baseline conditions. Then, azodopa was administered with a standard 20 μl pipette at a 3 μM concentration in 10 μl volume. After the administration, the recordings started again and continued for 10 more minutes. After the experiments ended, the mice were euthanized.

Photoswitching experiments. Azodopa was photoswitched with 365 nm light from two LEDs controlled by the computer via an Arduino board (yellow arrow, Figure 4a in the main article). This allowed a precise control of the illumination periods (one-minute ON cycles) that were timestamped to the Open Ephys recording file (purple arrow, Figure 4a in the main article; results of illumination in Supplementary Figures S17-S19). In two mice, the two LEDs were placed on top of their heads so that light was directed towards the craniotomy for illumination with 365 nm light. The Arduino UNO board turned on and off the two LEDs and sent analogical triggers to the Open Ephys data acquisition system that included them as timestamps in the electrophysiological recordings. The illumination patterns consisted in one-minute ON one-minute OFF cycles. In these experiments, we first performed controls with saline, where 0.5 ml of saline were administered into the pool via an infusion pump at 0.5 ml per minute. Subsequently, the experiment was repeated with 0.5 ml 3 μM azodopa. We conducted continuous recordings that included a 5 minute baseline and 12 minute epochs after saline or azodopa administration with ON/OFF illumination cycles of UV light. The first illumination cycle started simultaneously to the drug administration (1 minute period). The goal was to inactivate azodopa while it accumulated in the pool. After the experiments ended, the mice were euthanized.

Data analysis. Recorded signals from each electrode were filtered offline to extract spiking activity and local field potentials (LFPs). Spiking activity was estimated by first subtracting the raw signal from each electrode with the mean signal of the 8 electrodes, which removed artifacts present in all the electrodes. Then, continuous signals were filtered between 450-6000 Hz with Python (Butterworth bandpass filter, order 2) and saved as NEX files. Spike sorting was performed with the Offline Sorter v4 software (Plexon Inc.). We first thresholded the signal and removed noise artifacts manually. Then, the K-means algorithm was used for automatic sorting of spikes to minimize any bias in the analyses. To obtain LFPs, signals were detrended and decimated to 1 kHz with custom-written scripts in Python. Spectrograms were constructed using the spectrogram function of the SciPy package (10 s windows, no overlap) and power was quantified with the multitaper power method of the spectral_connectivity package in Python. The taper parameters chosen for the analyses were: time-half-bandwidth product = 5, 9 tapers.

Statistical analysis. The firing rate of individual neurons was compared between baseline and azodopa and between saline and azodopa with an unpaired *t*-test (baseline vs azodopa, n = 44 vs 43 neurons; saline vs azodopa, n = 57 vs 67 neurons). For each electrode, the mean firing rate of all neurons and LFP power (1 to 10 Hz) were compared between conditions with a paired *t*-test (n = 2 mice, 8 electrodes per mouse). In the photoswitching experiments, the firing rates of individual neurons and LFP power were compared between the light and dark cycles with a paired *t*-test. We omitted the first light cycle (when saline or azodopa were injected) and compared the following 5 light cycles with the prior 5 dark cycles. We identified neurons that

fired more in the dark than during light cycles (dark-ON neurons) and neurons with the opposite pattern (8 light-ON neurons) in Mouse 3. Statistical analyses were conducted with the GraphPad Prism 6 statistical package.

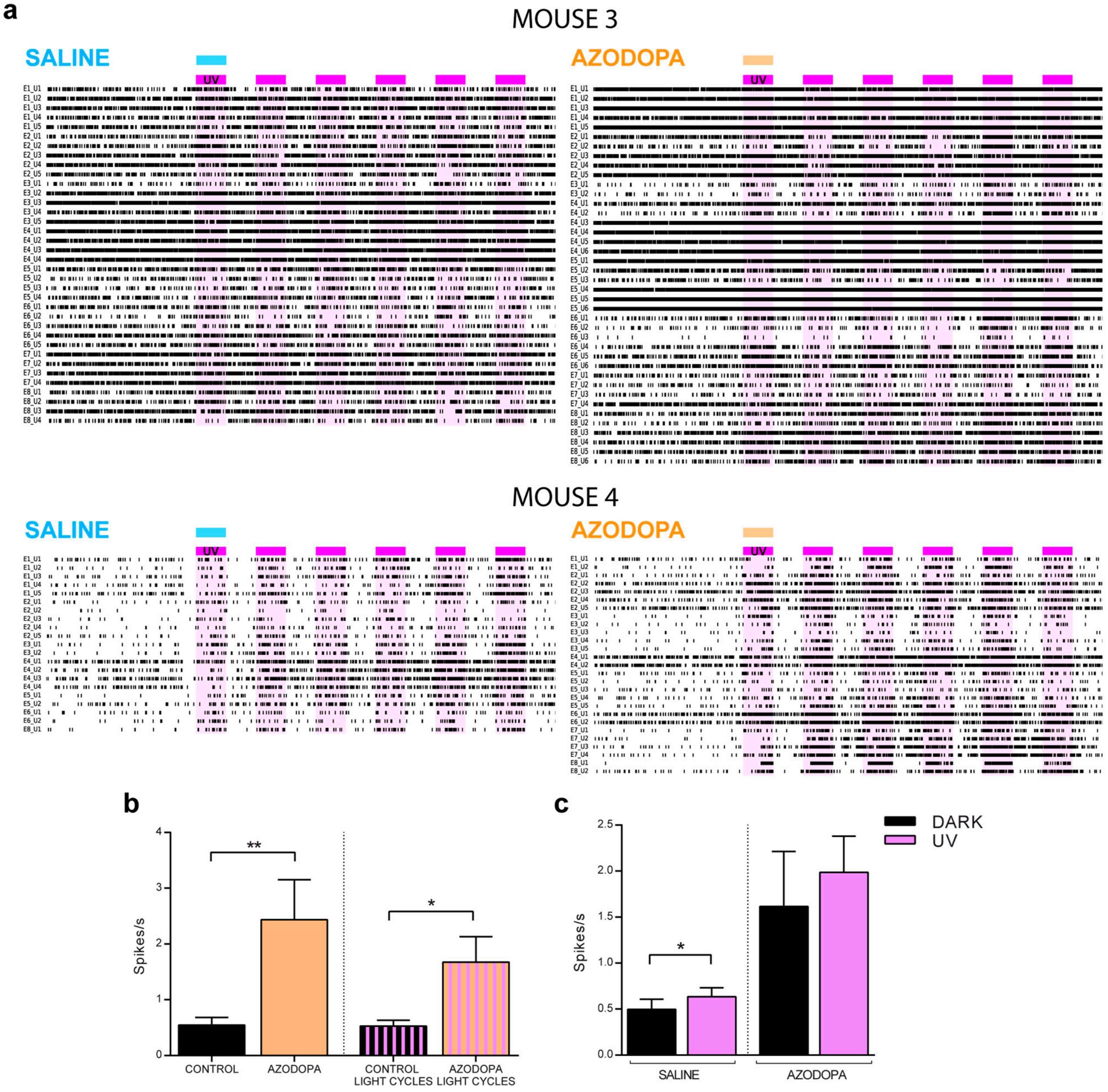

Figure S17. Effect of azodopa and illumination on spiking activity *in vivo*. (a) Raster plots of spiking activity in two mice anesthetized with isoflurane. Each row depicts the spiking activity of a single neuron (unit), each tick representing an action potential. We used arrays of 8 electrodes (octrodes) in each animal from which several units could be recorded. Neurons are labeled by their electrode number (E1 to E8). Six one-minute cycles of UV light are also shown (purple lines). Saline (0.5 ml) and azodopa (3 μM in 0.5 ml) were administered with an infusion pump during the first light cycle (blue and orange lines). Individual neurons in the secondary motor cortex recorded after the injection of saline and azodopa were isolated offline with the Offline Sorter software. Notice that different neurons, particularly in Mouse 3, respond to illumination with opposite firing patterns, some increasing their spiking activity (light-ON pattern) while others decreasing it (dark-ON pattern; see also Figure S18 below). (b) As in Mouse 1 and Mouse 2 in the dark (solid bars on the left, data from Figure 4 in the main text), azodopa increased the firing rate of neurons with illumination in Mouse 3 and Mouse 4 (striped bars on the right, n = 57 neurons after saline *vs* 67 neurons after azodopa in two mice; mean firing rate per electrode, n = 15 channels, paired *t*-test, *p = 0.0246). This increase was not significantly affected by the application of light cycles. (c) After saline, the averaged spiking rate was higher during the light than the dark cycles. After azodopa application this tendency in the averaged firing rate was maintained but not significant differences were obtained due to the cancellation of the opposing responses to light and darkness of individual neurons (see selected traces in Figure S18).

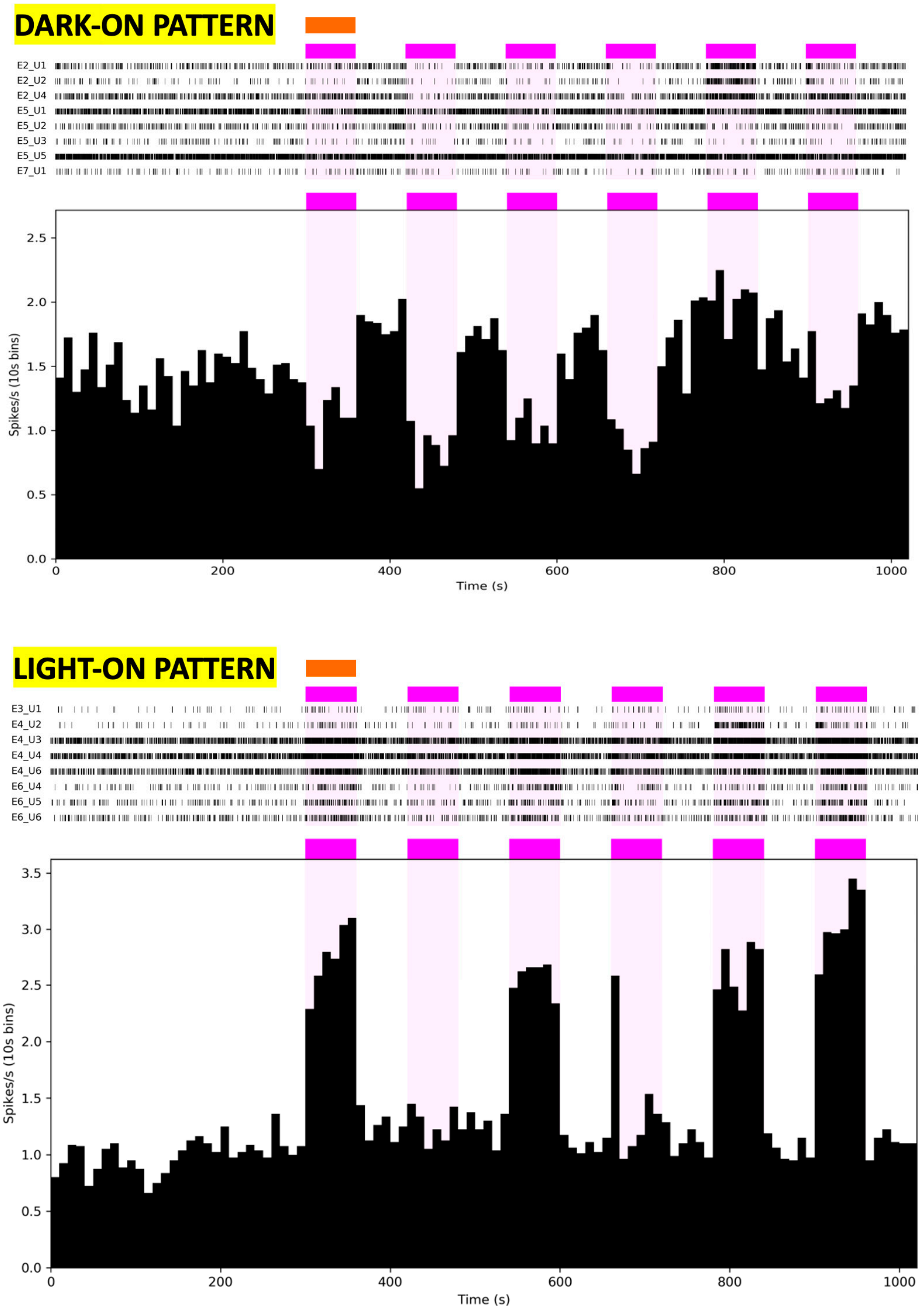

Figure S18. Individual neurons in the same mouse display opposite photoswitching patterns of spiking activity with azodopa. In Mouse 3, upon application of azodopa neurons can respond to light with opposite firing patterns, some decreasing their spiking activity during the light cycles (DARK-ON pattern, 8 pooled recordings) and others increasing it during the same cycles (LIGHT-ON pattern, 8 pooled recordings).

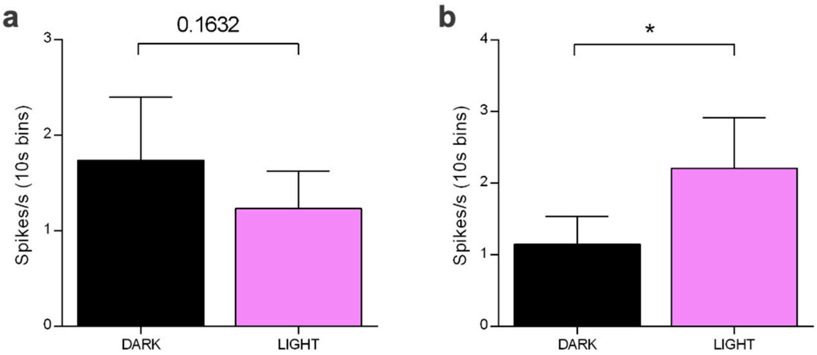

Figure S19. Photoswitching of spiking activity *in vivo* with azodopa. Quantification of the firing rate of (a) 8 neurons that decrease their spiking activity during the light cycles (dark-ON pattern) and (b) 8 neurons that increase their spiking activity during the light cycles (light-ON pattern). All neurons were recorded in Mouse 3. We compared the average spiking activity during the first 5 cycles of illumination (omitting the first cycle when azodopa was injected) with their previous 5 dark cycles (paired *t*-test; * p = 0.013).

9. Azodopa Resistance to Photodegradation

We verified in two ways that azodopa does not undergo significant irreversible photodegradation under the assay conditions, i.e. that the efficacy of the *trans* isomer can be maintained unaltered even after prolonged exposure to 365 nm light: (a) we analyzed a sample of azodopa by HPLC-PDA-MS before and after illumination (3 min and 60 min) (Figure S20), and (b) we compared in calcium imaging experiments the efficacy in the dark of a sample of azodopa that was pre-illuminated with 365 nm light for 60 min (named azodopa-60 for simplicity) with another sample of azodopa from the same batch but never exposed to light (Figure S21).

We did not detect any significant variation in the composition of the sample by HPLC-PDA-MS. Moreover, the efficacy in the dark of azodopa-60 was statistically not different from the efficacy of the non-illuminated sample.

HPLC-PDA

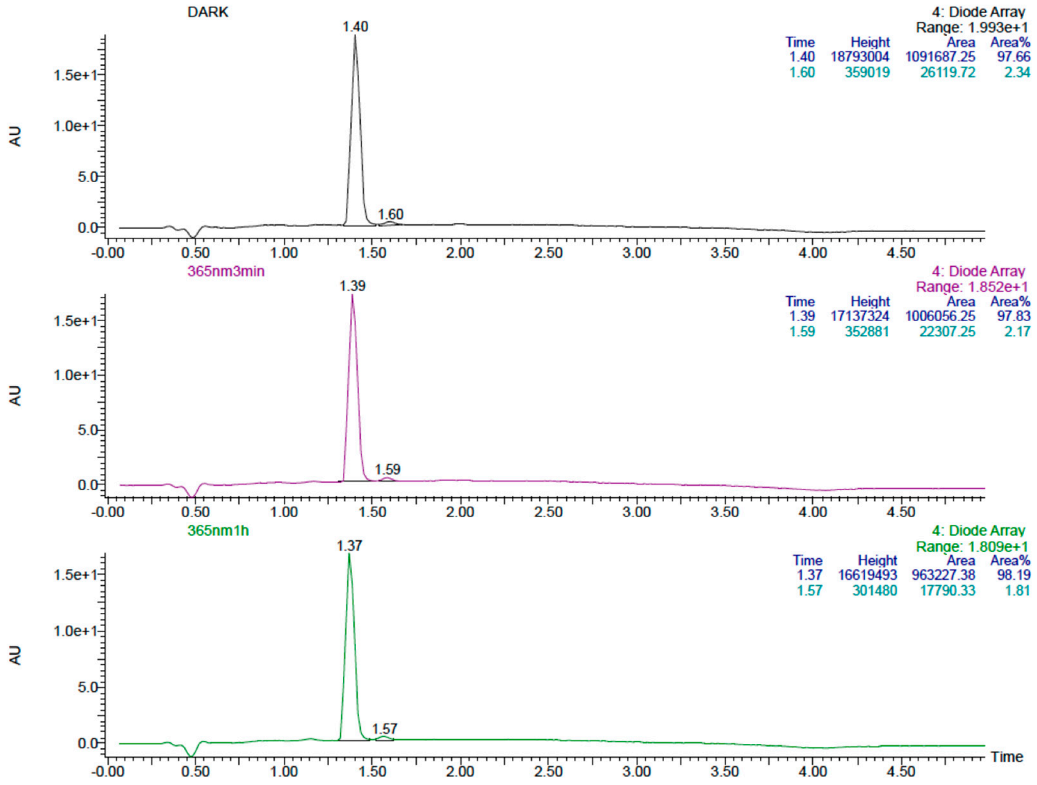

MS

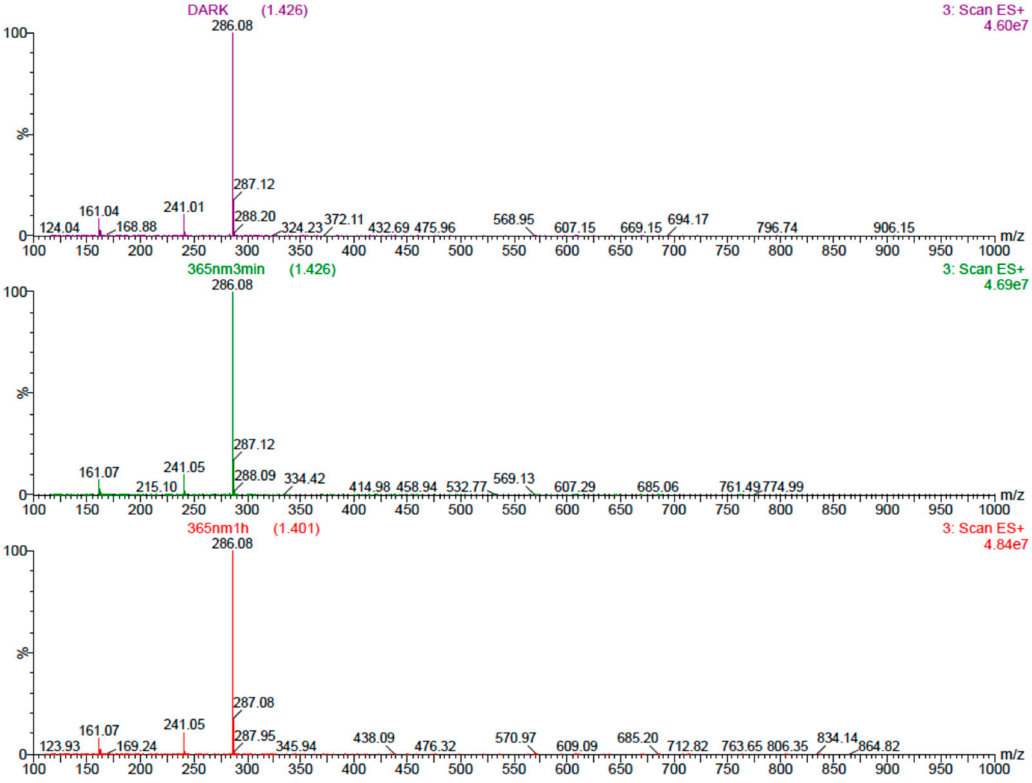

Figure S20. HPLC-PDA chromatograms and corresponding mass spectra of azodopa (50 µM) before and after illumination with a Vilber Lourmat UV Lamp (365 nm, 6 W) for 3 min and 60 min (top: dark; middle: 365 nm, 3 min; bottom: 365 nm, 60 min). No significant variations in the composition of the sample were detected.

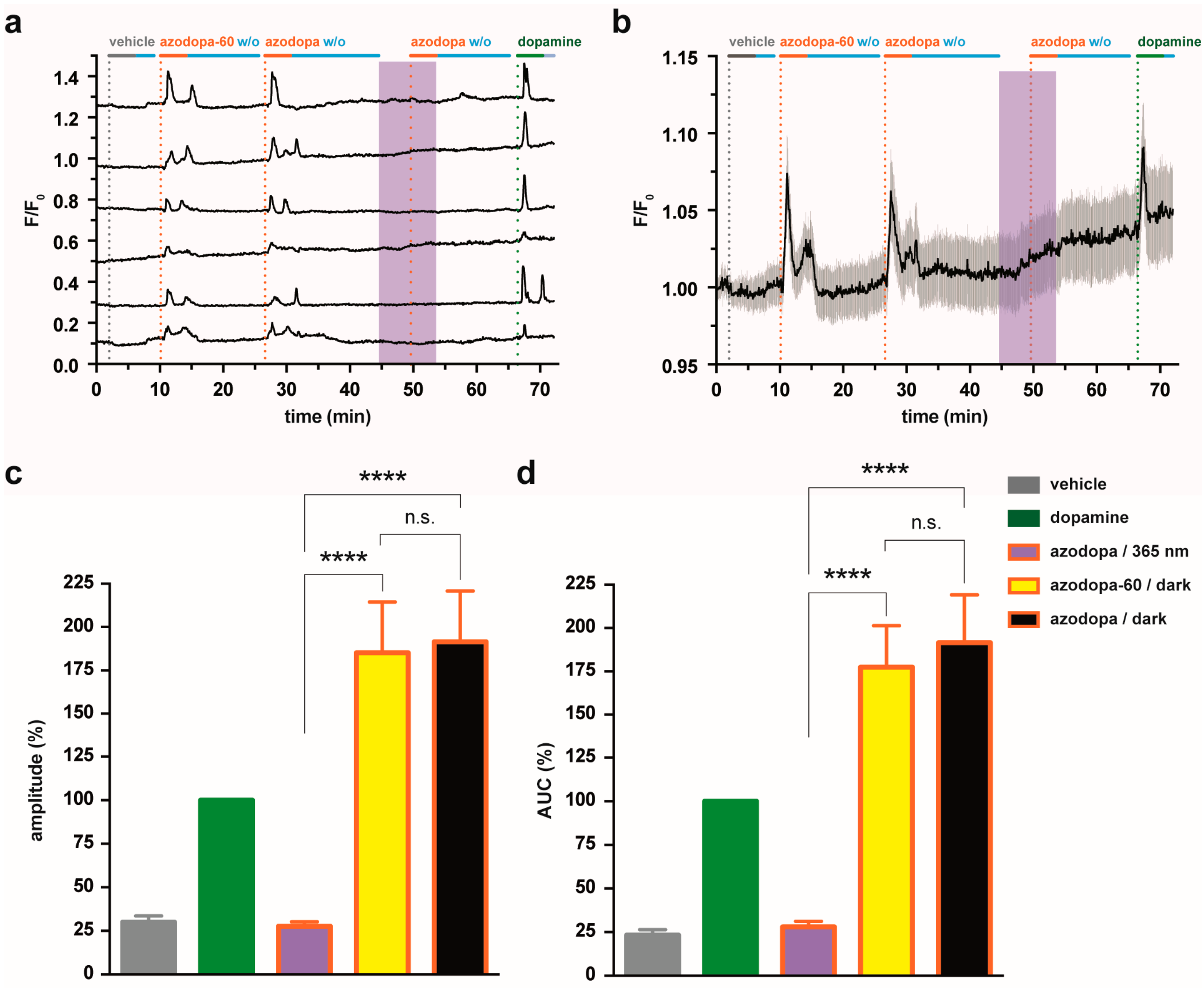

Figure S21. *trans*-Azodopa maintains its efficacy in calcium imaging experiments also after prolonged pre-exposure to ultraviolet light. Real-time calcium imaging response in HEK-293T cells co-expressing D_1 receptors and R-GECO1 as calcium indicator. (a) Single traces from 6 representative cells. (b) Averaged traces from 21 cells. Shadow represents '± S.E.M.'. Traces were recorded upon direct application of azodopa (200 µM, orange bars) in the dark (white areas) and under illumination (purple area). Azodopa-60 stands for "pre-irradiated azodopa" (Vilber Lourmat UV Lamp, 365 nm, 6 W, 60 min). Gray and green bars indicate the application of vehicle (control) and dopamine (reference agonist, 50 µM), respectively. Light blue bars indicate wash-out periods. Two values of the calcium responses generated by azodopa were calculated (Origin 8 software) and compared: the peak *amplitude* ΔF/F0 (c), calculated as the difference between the maximal and the minimal intensity of each response (**** $p < 0.0001$ for azodopa/365 nm *vs* azodopa-60/dark; **** $p < 0.0001$ for azodopa/365 nm *vs* azodopa/dark), and the area under the curve (AUC) (d), calculated as the integral over the entire application time of vehicle or drugs (**** $p < 0.0001$ azodopa/365 nm *vs* azodopa-60/dark; **** $p < 0.0001$ for azodopa/365 nm *vs* azodopa/dark). Data are mean ± S.E.M. (n = 34 cells from 3 independent experiments). Data were normalized over the maximum response obtained with the saturating concentration of dopamine (50 µM) and were analyzed by one-way ANOVA followed by Tukey's post-hoc test for statistical significance. All statistical analyses were performed with GraphPad Prism 6. As shown, no statistical difference was observed between azodopa and azodopa-60 neither in amplitude nor in AUC of the calcium responses generated.

10. Recovery of Normal Swimming Behavior of Zebrafish Larvae after Washout

We demonstrated that zebrafish larvae, previously treated with azodopa 100 µM and exposed to dark-light cycles, recover normal swimming behavior after washout (Figure S22, see caption for details). After the first experiment (panel a), the fish were transferred and maintained in fresh water for more than 1 hour, and the motility experiment was repeated using the same protocol of illumination (panel b). As shown in the figure, the two groups of fish displayed comparable swimming activity and reactivity to illumination after washout (panel b), independently if they had been previously exposed to azodopa or control solution. Moreover, all the larvae, kept in fresh water, were still alive after about 48 hours.

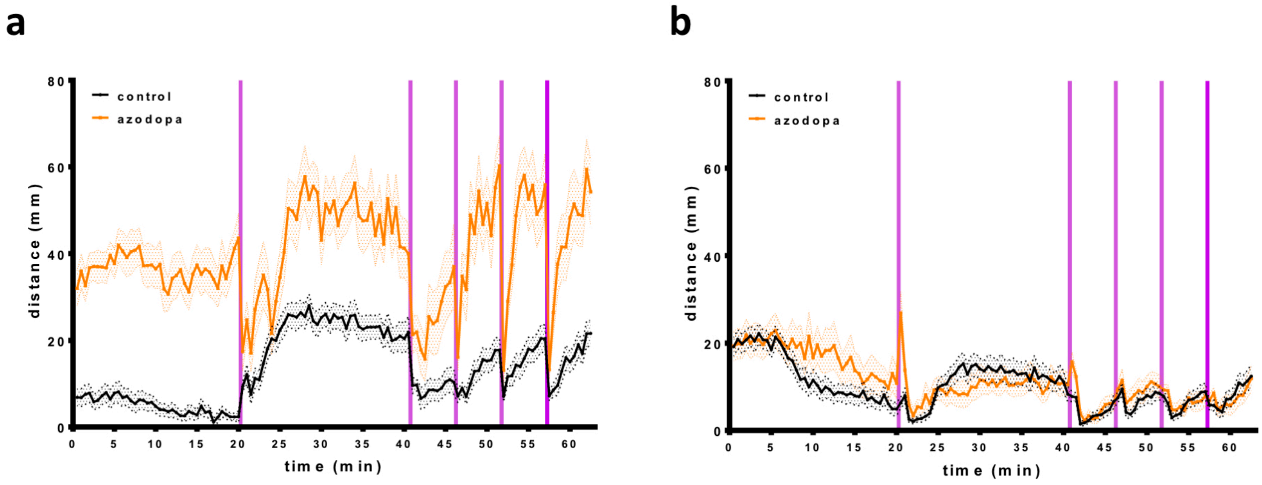

Figure S22. Recovery of normal swimming behavior of zebrafish larvae after washout. (a) Swimming activity (distance/time) in larvae exposed to vehicle (control, gray line) or 100 µM azodopa (treatment, orange line) in the dark (white areas) or under illumination with 365 nm light (purple bars), following the same experimental procedure and illumination protocol shown in Figure 3. (b) Swimming activity (distance/time) in larvae previously exposed to vehicle (control, gray line) or 100 µM azodopa (treatment, orange line) after washout. Washout procedure: fish were washed in fresh water (5 ml × 5 times, in a period of >1 hour) and then added to a new 96-well plate; each individual was placed into the same well position as in the first experiment; before starting the second experiment, all larvae were disturbed by adding 100 µl of water to each well in order to reproduce the same distress as in the first experiment (vehicle or treatment solution addition). Data are mean ± S.E.M. (n = 24 individuals/group).

11. Additional References

1. Casadó, V.; Cortés, A.; Ciruela, F.; Mallol, J.; Ferré, S.; Lluis, C.; Canela, E.I.; Franco, R. Old and new ways to calculate the affinity of agonists and antagonists interacting with G-protein-coupled monomeric and dimeric receptors: the receptor-dimer cooperativity index. *Pharmacol. Ther.* **2007**, *116*, 343–354.
2. Ferré, S.; Casadó, V.; Devi, L.A.; Filizola, M.; Jockers, R.; Lohse, M.J.; Milligan, G.; Pin, J.P.; Guitart, X. G protein-coupled receptor oligomerization revisited: functional and pharmacological perspectives. *Pharmacol. Rev.* **2014**, *66*, 413–434.
3. Casadó, V.; Ferrada, C.; Bonaventura, J.; Gracia, E.; Mallol, J.; Canela, E.I.; Lluís, C.; Cortés, A.; Franco, R. Useful pharmacological parameters for G-protein-coupled receptor homodimers obtained from competition experiments. Agonist-antagonist binding modulation. *Biochem. Pharmacol.* **2009**, *78*, 1456–1463.
4. Ferrada, C.; Moreno, E.; Casadó, V.; Bongers, G.; Cortés, A.; Mallol, J.; Canela, E.I.; Leurs, R.; Ferré, S.; Lluís, C.; Franco, R. Marked changes in signal transduction upon heteromerization of dopamine D1 and histamine H3 receptors. *Br. J. Pharmacol.* **2009**, *157*, 64–75.
5. Taylor, S.; Chen, J.; Luo, J.; Hitchcock, P. Light-induced photoreceptor degeneration in the retina of the zebrafish. *Methods Mol. Biol.* **2012**, *884*, 247–254.
